# Computational modeling suggests impaired interactions between NKX2.5 and GATA4 in individuals carrying a novel pathogenic D16N NKX2.5 mutation

**DOI:** 10.18632/oncotarget.24459

**Published:** 2018-02-09

**Authors:** Saidulu Mattapally, Mrityunjay Singh, Kona Samba Murthy, Shailendra Asthana, Sanjay K. Banerjee

**Affiliations:** ^1^ Division of Medicinal Chemistry and Pharmacology, CSIR-Indian Institute of Chemical Technology, Hyderabad 500007, India; ^2^ Drug Discovery Research Center (DDRC), Translational Health Science and Technology Institute (THSTI), Faridabad, Haryana 121001, India; ^3^ Innova Children’s Heart Hospital, Tarnaka, Hyderabad 500017, India

**Keywords:** molecular dynamics simulation, computational modeling, protein-protein interaction, pathogenic mutation, congenital heart disease

## Abstract

*NKX2.5*, a homeobox containing gene, plays an important role in embryonic heart development and associated mutations are linked with various cardiac abnormalities. We sequenced the *NKX2.5* gene in 100 congenital heart disease (CHD) patients and 200 controls. Our analysis revealed a total of 7 mutations, 3 in intronic region, 3 in coding region and 1 in 3’ UTR. Of the above mutations, one mutation was found to be associated with tetralogy of fallot (TOF) and two (rs2277923 and a novel mutation, D16N) were strongly associated with VSD. A novel missense mutation, D16N (p-value =0.009744), located in the tinman (TN) region and associated with ventricular septal defect (VSD), is the most significant findings of this study. Computational analysis revealed that D16N mutation is pathogenic in nature. Through the molecular modeling, docking and molecular dynamics simulation studies, we have identified the location of mutant D16N in *NKX2.5* and its interaction map with other partners at the atomic level. We found NKX2.5-GATA4 complex is stable, however, in case of mutant we observed significant conformational changes and loss of key polar interactions, which might be a cause of the pathogenic behavior. This study underscores the structural basis of D16N pathogenic mutation in the regulation of *NKX2.5* and how this mutation renders the structural-functional divergence that possibly leading towards the diseased state.

## INTRODUCTION

*NKX2.5* is one of the transcription factors plays an important role in heart development. It is a cardiac specific homeobox gene and acts as an early marker gene for heart field development. *NKX2.5* has been mapped to chromosome 5q31.1 and has two exons [1]. Similar to other members of the nucleotide kinase (NK)-2 class viz. *NKX2.1, NKX2.2, NKX2.3, NKX2.4 and NKX2.6*, *NKX2.5* contains three highly-conserved regions: the homeo-domain, the tinman (TN) domain and the NK2 (unique to NK2 class proteins) domain [2]. The homeo-domain is a highly-conserved DNA-binding domain located in the core, while the short TN domain is found at the N-terminal region of most NK-2 proteins [3]. *NKX2.5* binds to the consensus sequence of the ANF promoter and interacts with other transcriptional factors such as; *GATA4* and *TBX5* [4–6]. During fetal heart development, mutation in *NKX2.5* gene leads to fetal cardiac structural morphogenesis, growth retardation, and embryonic lethality. *NKX2.5* knockout mice died from cardiac malformations [7, 8]. Mutation of *NKX2.5* in zebrafish develops embryos with diminutive ventricular and bulbous atrial chambers [9]. Till date, more than 40 heterozygous *NKX2.5* germline mutations associated with CHD patients were reported [10–13]. However, the mechanisms by which mutations cause cardiac defects remain largely unknown.

CHD is a multifactorial disease in which genetic and environmental factors play an important role to develop the disease. Genetic events such as mutations and chromosomal aberrations are responsible for CHD [14, 15]. Several studies reported that *NKX2.5* gene mutations cause different types of CHDs like atrial septal defect (ASD), ventricular septal defects (VSD), tetralogy of fallot (TOF) and single ventricle (SV). Recent studies reported that the prevalence of *NKX2.5* mutations is about 1–4% in sporadic patients with ASD [11]. VSD occurs in approximately 50% of all children with CHD, and accounts for 14 to 16% of the defects that require an invasive procedure within the first year of life [16, 17]. VSD can occur alone or with other cardiac anomalies, such as ASD, down-syndrome, or TOF. Compared to other countries, the prevalence of CHD in India is considerably higher [18]. However, very few studies on atomic level at structural basis have been conducted to find the mutational causes of CHD.

We identified a novel D16N mutation in *NKX2.5* by screening 100 South Indian CHD patients. We are elucidating the molecular basis as how this mutation becomes pathogenic and cause CHD through protein-protein interaction study. The structural localization of D16N mutation and their atomic level resolution showed as how this mutation alters the architecture of functional protein-protein and/or protein-DNA interactions. Additionally, we also tried to explore the structural understanding of disease associated reported mutations. It is known that *GATA4*-*NKX2.5* partnership may represent a paradigm for transcription factor interaction during organogenesis [5]; however, the mode of interaction has not been reported yet. The protein-protein interaction between *NKX2.5* and *GATA4* provides the structural insights as how D16N mutation perturbed the structural-functional relationship, which might be responsible for pathogenicity.

## RESULTS

### Clinical evaluation and mutations in *NKX2.5* gene

We collected sample from a total of 100 CHD patients. Sample selection was based on two criteria, first, was CHD types (ASD, VSD, TOF, and SV) and second, was demographics, i.e. selection of the study population based on geographical location, speaking the Dravidian language (Dravidians) and living in southern India. The percentage of CHD patients belong to different categories are as follows; ASD: 33%, VSD: 32%, TOF: 32%, SV: 3%. Age of all CHD patients is ranging from 0.35 to 10.79 years. However, maximum number of CHD patients used for this study was <5 years [19]. Two hundred individuals having no CHD or any family history of CHD or heart disease were included in this study as control. The study conforms to the principles outlined in the Declaration of Helsinki and was approved by the research advisory committee and institutional ethical committee of Innova Children’s Heart Hospital, Hyderabad.

**Figure 1:**
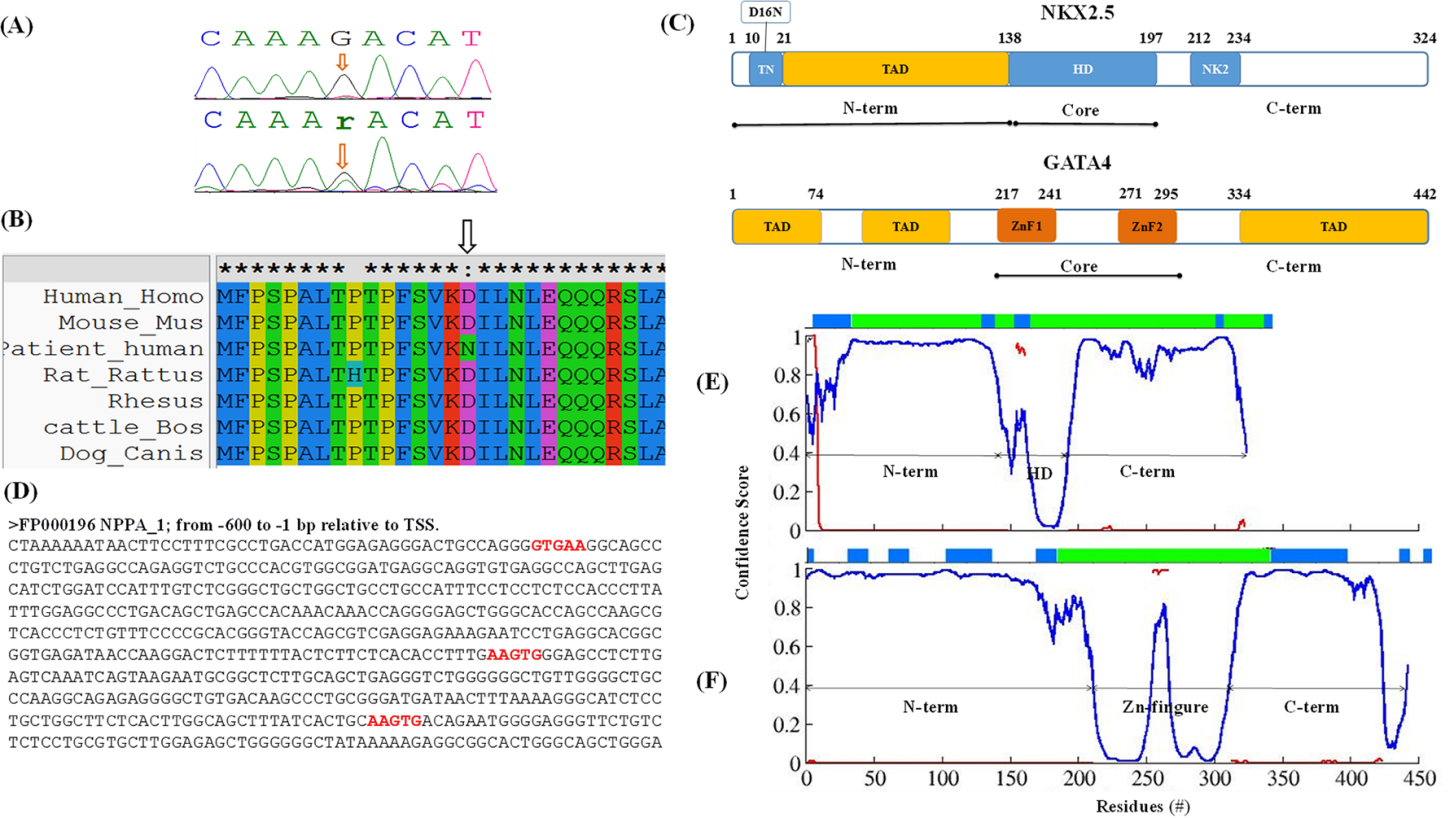
**(A)** DNA sequencing electropherogram showing wild type (upper panel) and heterozygous (G → A) mutation (lower panel) in *NKX2.5* coding region (arrows), mutation D16N, **(B)** Multiple amino acid sequence alignment of different species shows the conservation of the mutated amino acid residue (amino acid marked) across species. **(C)** Schematic diagram of *NKX2.5* and *GATA4. NKX2.5*: structure/architecture depicting the location of a novel mutation D16N; N-term, amino-terminal region; TN, tin-man domain; HD, homeo-domain; NK2, nucleotide kinase domain-2; C-term, carboxy-terminal region. *GATA4*: TAD, transcription activation domain; two Zn finger motifs, ZnF1 (BR2) and ZnF2 (BR1). Three regions, N-term (amino-terminal region); core and C-term (carboxy-terminal region) are mentioned. The molecular modeling studies were conducted on N-term + core in case of *NKX2.5*, and only core in *GATA4*, is highlighted by horizontal black lines. **(D)** Sequence of Atrial natriuretic factor (ANF) promoter region from −600bp to −1bp. The motifs shown in red color are potential binding motifs for human *NKX2.5* in human ANF promoter, identified from literature. In panels **(E** and **F)** Structural disorder and globularity predictions: the disorder regions (confidence score higher than 0.5) are shown by blue lines and protein binding region shown by red lines. Confidence score shown in Y-axis, and residues in X-axis. Globularity prediction is shown as horizontal bar at the top, disordered region (in blue), ordered region (in green) and the gaps represent coil regions, (E) *NKX2.5* and (F) *GATA4.*

**Figure 2:**
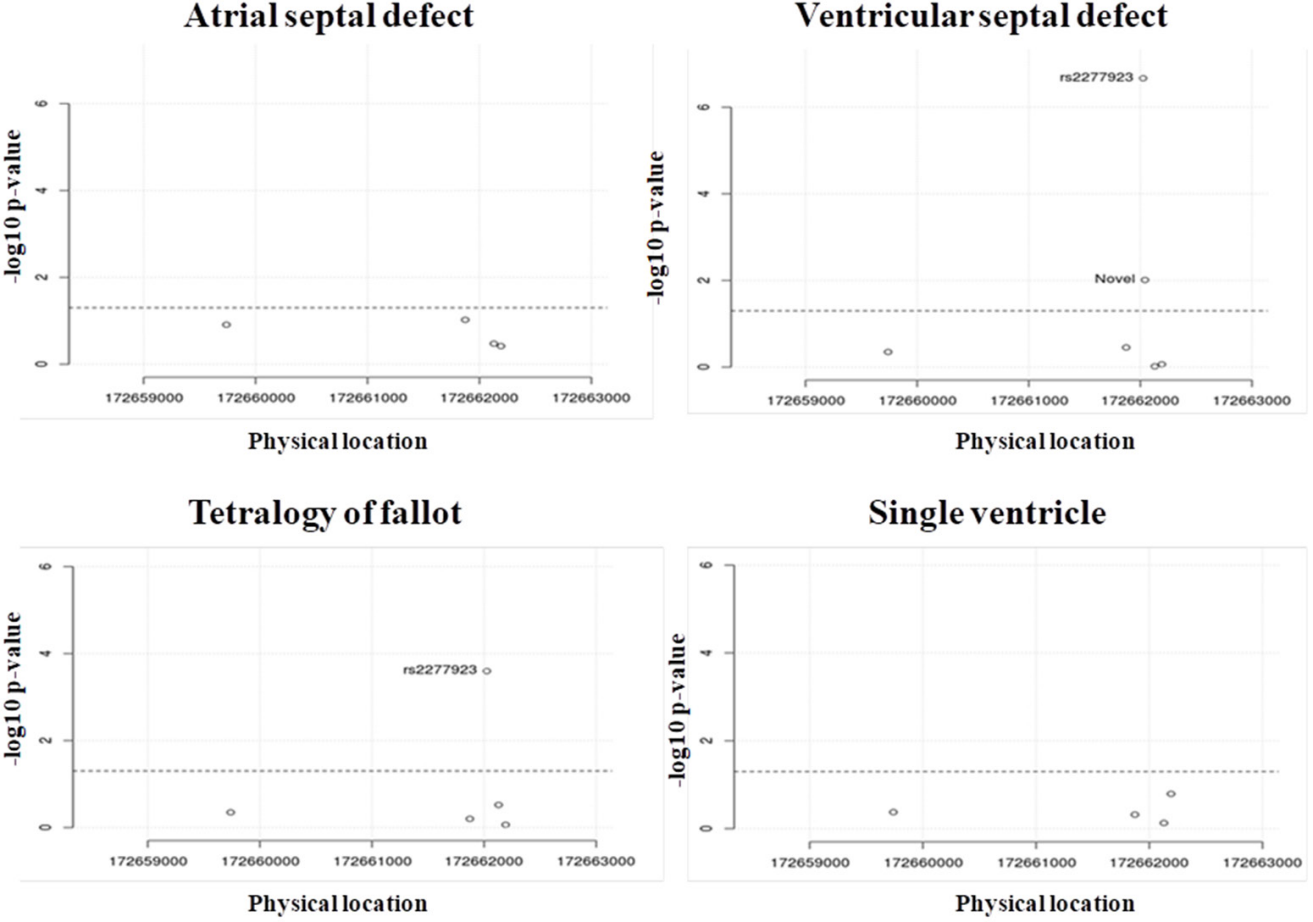
Chi-square P-value of variations found in NKX2.5 of all types of CHD **(A)** atrial septal defect (ASD), **(B)** ventricular septal defect (VSD), **(C)** tetralogy of fallot (TOF) and **(D)** single ventricle (SV).

Our analysis revealed a total of 7 mutations, out of which 4 mutations were in intronic regions, 2 mutations were in exonic region (one missense and one synonymous mutation) and 1 mutation was in 3’ UTR (Table 1). We classified the samples based on phenotype: ASD, VSD, TOF, and SV and removed one marker, which were not following Hardy-Weinberg equilibrium in ethnically matched control samples. This marker was rs703752 (HWE p-value = 0.003877) (Supplementary Figure 1). *In-silico* analysis showed that one missense mutation was conserved and predicted to be pathogenic (Figure 1A and 1B). Further, we performed Chi-square analysis for finding the statistical significance. In TOF, rs2277923 (p-value = 0.0002528) was associated with the disease. In case of VSD, 2 mutations have shown strong association. They are rs2277923 (p-value = 2.142e-07) and novel D16N (p-value = 0.009744) (http://www.ashg.org/2014meeting/abstracts/fulltext/f140121487.htm; dbSNP reference numbers: NM_001166175.1:c.46G>A, NM_001166176.1: c.46G>A, NM_004387.3: c.46G>A, XM_017009071.1: c.46G>A) (Figure 2, Table 2).

**Table 1:** List of all *NKX2.5* mutations identified in the present study

S. No.	dbSNP-ID	Physical position (hg19)	Nucleotide variation	Mutation type	AA change	Observe hetero.	Expected hetero.	P-value: HWE
1	rs703752	172659511	G>T	3’UTR	-	0.03	0.04875	0.003877
2	Novel-Intronic	172659740	C>G	Intronic	-	0.155	0.143	0.612
3	CM086533	172661873	G>A	Intronic	-	0.17	0.1556	0.3696
4	rs2277923	172662024	G>A	Synonymous	E63E	0	0	1
5	Novel-Exonic	172662041	G>A	Missense	D16N	0	0	1
6	rs3729937	172662129	C>T	Intronic	-	0.035	0.03439	1
7	rs77083308	172662192	G>A	Intronic	-	0.09	0.08595	1

**Table 2:** Significance test for *NKX2.5* mutations

Disease	CHR	db-SNP-ID	Physicalposition (hg19)	Minor allele	Frequency of minor allele in cases	Frequency of minor allele in control	Major allele	Chi-sq. value	P-value	OR
ASD	5	Novel-Intronic	172659740	A	0.01923	0.0775	G	2.375	0.1233	0.2334
	5	CM086533	172661873	A	0.01923	0.085	G	2.786	9.51E-02	0.2111
	5	rs2277923	172662024	A	0	0	G	NA	NA	NA
	5	Novel-Exonic	172662041	G	0	0	C	NA	NA	NA
	5	rs3729937	172662129	T	0	0.0175	C	9.24E-01	0.3363	0
	5	rs77083308	172662192	A	0.01923	0.045	G	0.7589	0.3837	0.4161
VSD	5	Novel-Intronic	172659740	A	0.05	0.0775	G	0.5764	0.4477	0.6265
	5	CM086533	172661873	A	0.05	0.085	G	0.8641	0.3526	0.5666
	***5***	***rs2277923***	***172662024***	***A***	***0.06667***	***0***	***G***	***26.9***	***2.14E-07***	***NA***
	***5***	***Novel-Exonic***	***172662041***	***G***	***0.01667***	***0***	***C***	***6.681***	***0.00974***	***NA***
	5	rs3729937	172662129	T	0.01667	0.0175	C	0.00212	0.9633	0.9516
	5	rs77083308	172662192	A	0.05	0.045	G	0.02994	0.8626	1.117
TOF	5	Novel-Intronic	172659740	A	0.05	0.0775	G	0.5764	0.4477	0.6265
	5	CM086533	172661873	A	0.06667	0.085	G	0.2314	0.6305	0.7689
	***5***	***rs2277923***	***172662024***	***A***	***0.03333***	***0***	***G***	***13.39***	***0.00025***	***NA***
	5	Novel-Exonic	172662041	G	0	0	C	NA	NA	NA
	5	rs3729937	172662129	T	0	0.0175	C	1.066	0.3018	0
	5	rs77083308	172662192	A	0.05	0.045	G	0.02994	0.8626	1.117
**SV**	5	Novel-Intronic	172659740	A	0.1667	0.0775	G	0.6473	0.4211	2.381
	5	CM086533	172661873	A	0.1667	0.085	G	0.5005	0.4793	2.153
	***5***	***rs2277923***	***172662024***	***A***	***0.1667***	***0***	***G***	***66.83***	***2.96E-16***	***NA***
	5	Novel-Exonic	172662041	G	0	0	C	NA	NA	NA
	5	rs3729937	172662129	T	0	0.0175	C	0.1068	0.7438	0
	5	rs77083308	172662192	A	0.1667	0.045	G	1.962	0.1613	4.244

### *In-silico* analysis of missense mutation

In the coding region, we found one missense mutations and one synonymous mutation in CHD patients. These two mutations are D16N and E63E, respectively. Multiple alignments of *NKX2.5* amino acid sequences from human, cattle, monkey, pig, dog, rat and mouse found that D16 is conserved during evolution (Figure 1B). To know the functional significance of missense mutations, we performed bioinformatics analysis of identified missense mutation using PMut (http://mmb.irbbarcelona.org/PMut/), SIFT (http://sift.bii.astar.edu.sg/), PROVEAN (http://provean.jcvi.org/seq_submit.php), PANTHER (http://www.pantherdb.org/tools/csnpScoreForm.jsp), PHD-SNP (http://snps.biofold.org/phd-snp/phd-snp.html), SNAP (https://www.rostlab.org/services/snap/) and PredictSNP (https://loschmidt.chemi.muni.cz/predictsnp1/). These tools predict whether an amino acid substitution or indel has an impact on the biological function of a protein or not.

The outcome of PMut, PROVEAN, SIFT, PANTHER, PHD-SNP, SNAP and PredictSNP strongly indicate that the D16N mutation is pathogenic (Table 3). Hence to explore the structural mechanistic aspect, how D16N mutation leads to the disease state, we performed the extensive computational structural dynamics study.

**Table 3:** Prediction of functional significance of the D16N *NKX2.5* mutation by using multiple computational programs

Name of software	D16N		Cut-off
	Prediction	Score	
PMut	Pathological	0.72 (86%)	0.5
SIFT	Pathological	0	0.05
PROVEAN	Pathological	-3.75	-2.5
PANTHER	Pathological	1037	450
PHD-SNP	Pathological	0.674	0.5
PredictSNP	Pathological	0.72%	0.50%
PolyPhen-2	Benign	0.029	0.5

### Structural localization of D16N in TN domain of *NKX2.5* and its interaction map

#### Molecular modeling

Structural localization of mutation is essential to relate its effects on protein functional phenotype. We carried out protein-DNA interaction, protein-protein interaction, and molecular dynamic simulation studies to know how D16N@*NKX2.5* mutation renders the protein resistant and paving its pathway towards the diseased state. We believe either it perturbs the interaction with ANF-242 promoter DNA or disturbing the association with other transcription factor viz. *GATA4, TBX5*. To answer this question, it is fundamental to determine, complete structure of *NKX2.5* specially N-term region and interaction pattern of the residue D16 with substrates like ANF-242 promoter (DNA), *GATA4* and *TBX5*. We performed our modeling studies in a systematic way as we constructed different sets of model complex systems: NKX2.5 (apo), NKX2.5-DNA, NKX2.5-GATA4, NKX2.5-TBX5, NKX2.5-DNA-GATA4, and NKX2.5-DNA-TBX5. To explore the effect of D16N we first evaluated the vicinity of D16 residue from the DNA and other substrate. Since the crystal structure of complete sequence of *NKX2.5* and *GATA4* has not been reported so far, except, the homeodomain (HD) of *NKX2.5* [6, 20] and Zn-finger domain of *GATA4* (PDB ID: 2M9W). Therefore, molecular modeling was carried out to model DNA (ANF-promoter), *NKX2.5* and *GATA4* complex assembly. A schematic diagram of *NKX2.5* and *GATA4* proteins depicting the structural domains and the location of the mutation D16N@*NKX2.5* detected in present study is delineated in Figure 1C.

#### Modeling of the DNA (ANF-242 promoter)

Human ANF promoter is the key, direct, and downstream target of *NKX2.5*, which involve in cardiac development. Each transcription factor binds to its own specific binding motif in DNA, and for each transcription factor, ANF promoter has many several me-too binding motifs [5, 6]. Therefore, it is interesting to identify the most likely binding motif.

Through JASPER tool [21] we predicted the binding site of transcription factor in promoter-DNA with the help of motif matrix model. We found 16 putative binding sites in ANF promoter region (Supplementary Table 1). Recently, Pradhan et. al. highlighted that *NKX2.5* binds preferentially in human ANF promoter DNA at “-242AAGTG” motif with TT at its 5′terminal (TTAAGTG). So, we search motif “AAGTG” in identified 16 NKE motifs. Out of 16, we found two motifs at position −242 and −80 matched with AAGTG motif (Figure 1D and S2B and, Supplementary Table 1). It is reported that ANF-80 site has weak binding affinity with *NKX2.5* than ANF-242 site [6]. Additionally, the ANF-242 binding region is also known for binding of other transcription factor like *GATA4*. It is well documented that interaction of *GATA4* necessary for activation of *NKX2.5* [5]. Due to the above-mentioned reasons, the ANF-242 was considered for model building steps.

#### Modeling of *NKX2.5*

**Figure 3:**
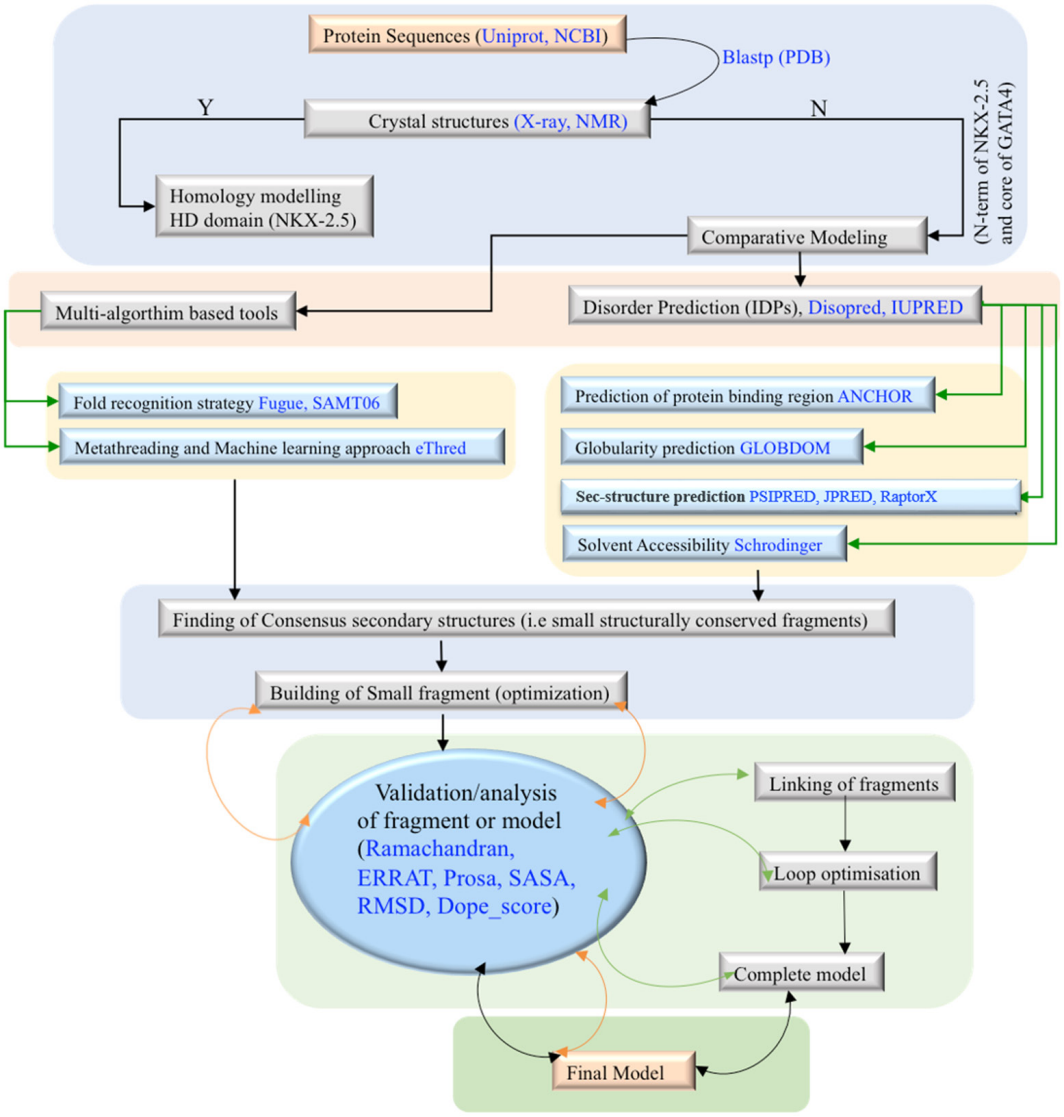
Scheme1: Flowchart of molecular modeling pipeline

In absence of crystal data of *NKX2.5* (except HD region) the dis-orderedness analysis followed [22] by globularity prediction [23] was performed to get the clues that how much *NKX2.5* is structured, intrinsically disordered protein (IDP) and complete disordered. It is a known fact that TFs are undergone disorder-to-order transitions upon binding to other substrates and/or partners protein [24, 25]. Similarly, several folded proteins regulate order-to-disorder transition to mediate their biological function/s [25–27]. Therefore, the information of binding regions (BRs), IDPs and completely disordered regions were used for the comparative model building of N-term region of *NKX2.5*. In the multi-step approach delineated as a scheme1: flowchart of molecular modeling pipeline (Figure 3), the quality assessment of models (fragments optimization and linking of fragments, and loop optimization) was verified through multi-round analysis via ramachandran plot [28], SASA [29], RMSD, ERRAT [30], ProSA_Zscore [31], Dope_Score [32] and secondary structure analysis. Final models were optimized through molecular dynamics simulations (see supplementary data for more details).

**Figure 4:**
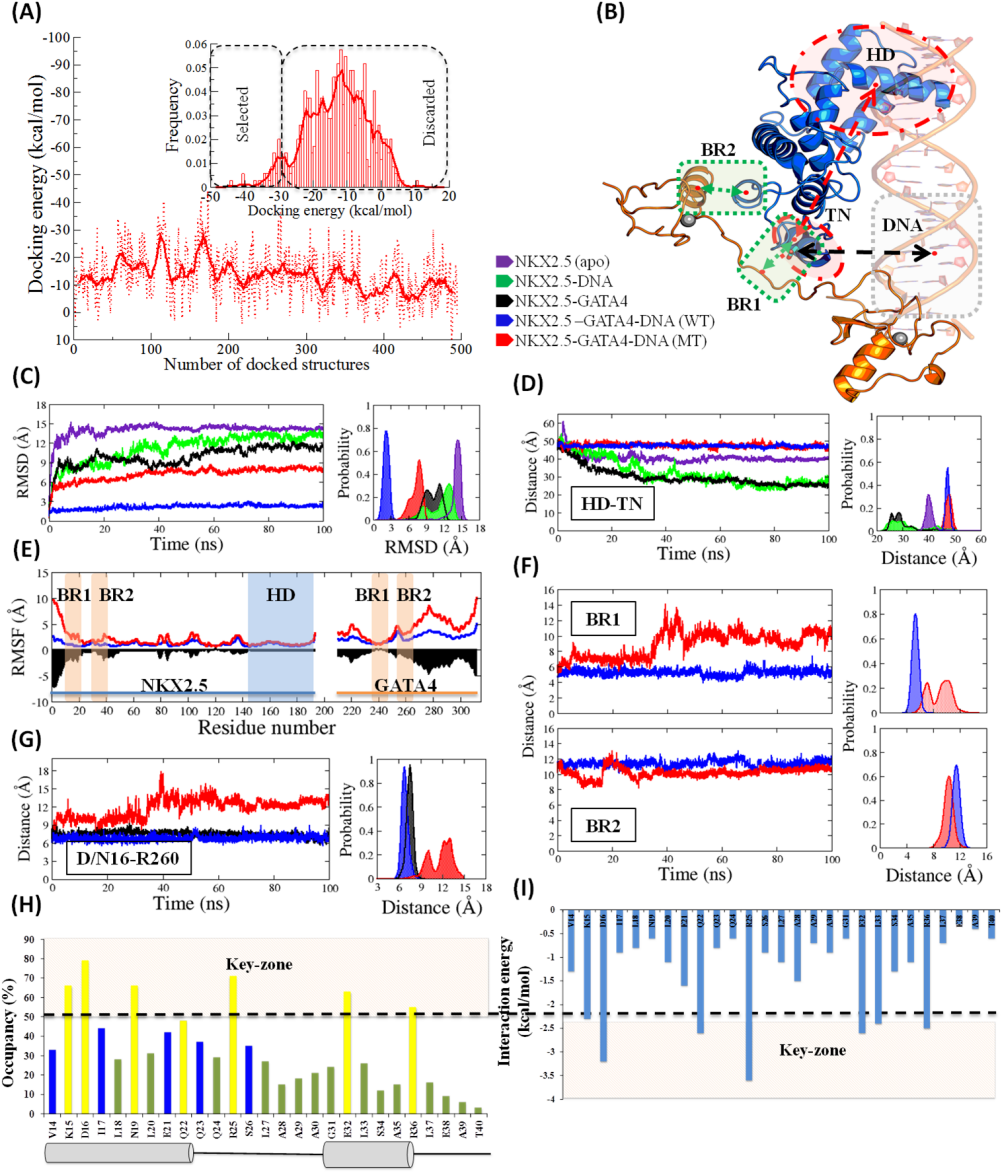
Molecular dynamics simulation analysis: **(A)** Selection of best-docked pose of *NKX2.5* and *GATA4* (complexes) from Protein-protein interface through PP-Docking. Histogram differentiates between zone of selected conformers and discarded conformers of complex. **(B)** Cartoon representation of NKX2.5 (D16)-DNA-GATA4 (WT) complex with highlighted domain movement (HD-TN, distance between center of mass of HD and TN domain of NKX2.5) in red colour, distance between TN@NKX2.5 and DNA in black colour and distance between NKX2.5 and GATA4 at binding region (BR1 and BR2) in green colour. In panels **(C to G)** the colour code for the MD systems are:*NKX2.5* (apo, in violet), *NKX2.5-* DNA (in green), *NKX2.5-GATA4* (in black), *NKX2.5*-DNA-*GATA4* (WT, in blue) and *NKX2.5*-DNA-*GATA4* (MT, in red). (C) The Root mean square deviation (RMSD, all backbone atoms) in coordinates as a function of the molecular dynamics simulation time. (D) The Domain movement between HD domain and TN domain throughout simulation. (E) RMSF value of Cɑ atoms comparing WT complex and MT complex simulations. (F) Distance analysis at BR1 and BR2 in WT and MT (G) Distance comparison between D/N16 and R260 in WT and MT complex system throughout simulation. **(H)** Occupancy of hydrogen bonds at BR1 site. The secondary structure helixes are shown by tubes and coils. **(I)** Residue wise interaction energy analysis (in kcal/mol).

Molecular modeling was performed separately for N-term and HD domains of *NKX2.5* through different approaches and then validated separate domains were concatenated to get the desired final models (Scheme 1). The concatenation is only continuing when it fulfilled the validation and optimization cut-offs and each step was verified through model analysis steps followed by the energy minimization and simulation through molecular dynamics (Scheme1, Figure 3). The results show that the final model of *NKX2.5* is stable during the dynamics from 20 to 100ns (Figure 4C). The closest to the average structure was extracted from last 80ns of the MD simulation and a Ramachandran plot was performed for model accuracy check (Supplementary Figure 3). The model was further evaluated by Prosa Z-score, which is −4.57 (4S0H), −4.54 (model HD@NKX2.5), −4.28 (model N-term@NKX2.5) and −4.0 (model complete NKX2.5 N-term+ HD part) (Supplementary Figure 4). The ERRAT values are 98.0 (4S0H), 94.8 (3RKQ) and 91.7 and (model: N-term+HD) (Supplementary Figure 5). These values are supported well with Ramachandran scores, which are 92.0% (4S0H), 97.8% (model HD), 86.7% (model N-term) and 89.7% (model: N-term+HD) (Supplementary Figure 3). The same steps were carried out for *GATA4*. See supplementary data for more details.

### Identification of disorder, binding and globular regions

The amino acid residue from 1 to 30 and 315 to 324 are moderately disordered (confidence score 0.6-0.8), residue 30 to 130 and 195 to 315 are highly disordered (confidence score >0.8), residue 130 to 140 are partial disordered (confidence score ∼ 0.6) and residue 140 to 195 are structured (confidence score < 0.5) (Figure 1E). DISOPRED used DISOPRED2 dynamic disorder prediction method provides accurate estimation of disorder prediction of around 93.1% [33].

The protein binding regions in intrinsic disorder proteins (IDPs) may undergo disorder-to-order transition during protein binding state and acquired specific secondary structure to execute their biological function [34]. Hence, we also checked the protein binding regions through ANCHOR [34] program. The prediction of binding regions is based on estimating the energy content in free and bound states, and identifying segments that are potentially sensitive to these changes. Identification of these segments is based on motif search algorithm. It was suggested that interaction with certain proteins or protein families are mediated through specific linear motifs. A growing number of such linear motifs are now being categorized through ELM server [35]. The anchor tool identified the three most prominent protein binding regions in *NKX2.5*: TN domain (at N-term), HD region, and C-term (Figure 1E). The α1 helix (residue 146-159) HD region of *NKX2.5* interacts with *TBX5* [20] while C-term of *NKX2.5* interacts with C-term region of *GATA4* [5]. Our models corroborate well with these finding, which confirmed that Binding Region (BR) prediction for model building was selected with high accuracy. We found in NKX2.5-GATA4 protein-protein interaction study that, the protein binding zone at region TN domain@*NKX2.5* interact with protein binding zone in region ZnF@*GATA4*.

Furthermore, we performed the globularity prediction to cross check the disorderedness findings, and binding regions of the *NKX2.5* (Figure 1E). DISOPRED data shows that residues 10 to 140 in *NKX2.5* are highly disorder (above the cutoff value 0.5), but the globularity prediction showed that residues 40-140 are globular and may have some linear motif. This result matched well with secondary structure prediction (Supplementary Figure 6) and protein binding motif outcomes. Similarly, in *GATA4* the region beyond the core domain residue 210-320 has no globular region and is completely disordered.

From the result of protein binding affinity, disorder tendency analysis and globularity prediction it is clear that although the region TN-domain in N-term and C-term of *NKX2.5* are disordered and missing in the reported crystal structures, but may gain secondary structure during the interaction. In *NKX2.5*, protein binding regions are presents at both N-term and C-term region. While C-term, is reported as an autorepressive domain and its interaction with C-term of *GATA4* might induce a conformational change that leads to the unmasking of *NKX2.5* activation domain [5]. Since the mutant D16N localizes at N-term and no interactions are yet described for the N-term region. Therefore, to explore the effect of D16N mutant in disease progression, only the N-term and HD regions were modeled for *NKX2.5* (region highlighted with black line in Figure 1C). Similarly, in the case of *GATA4*, protein binding region ware found between ZnF motif and extended C-term, and this also matched with globularity prediction and consensus secondary structure prediction data (Figure 1F). So, for the present study, only ZnF domain and extended C-term was modelled and shown for the *GATA4* (region highlighted with black line in Figure 1C).

### Protein-DNA Docking: DNA binds to HD domain

**Figure 5:**
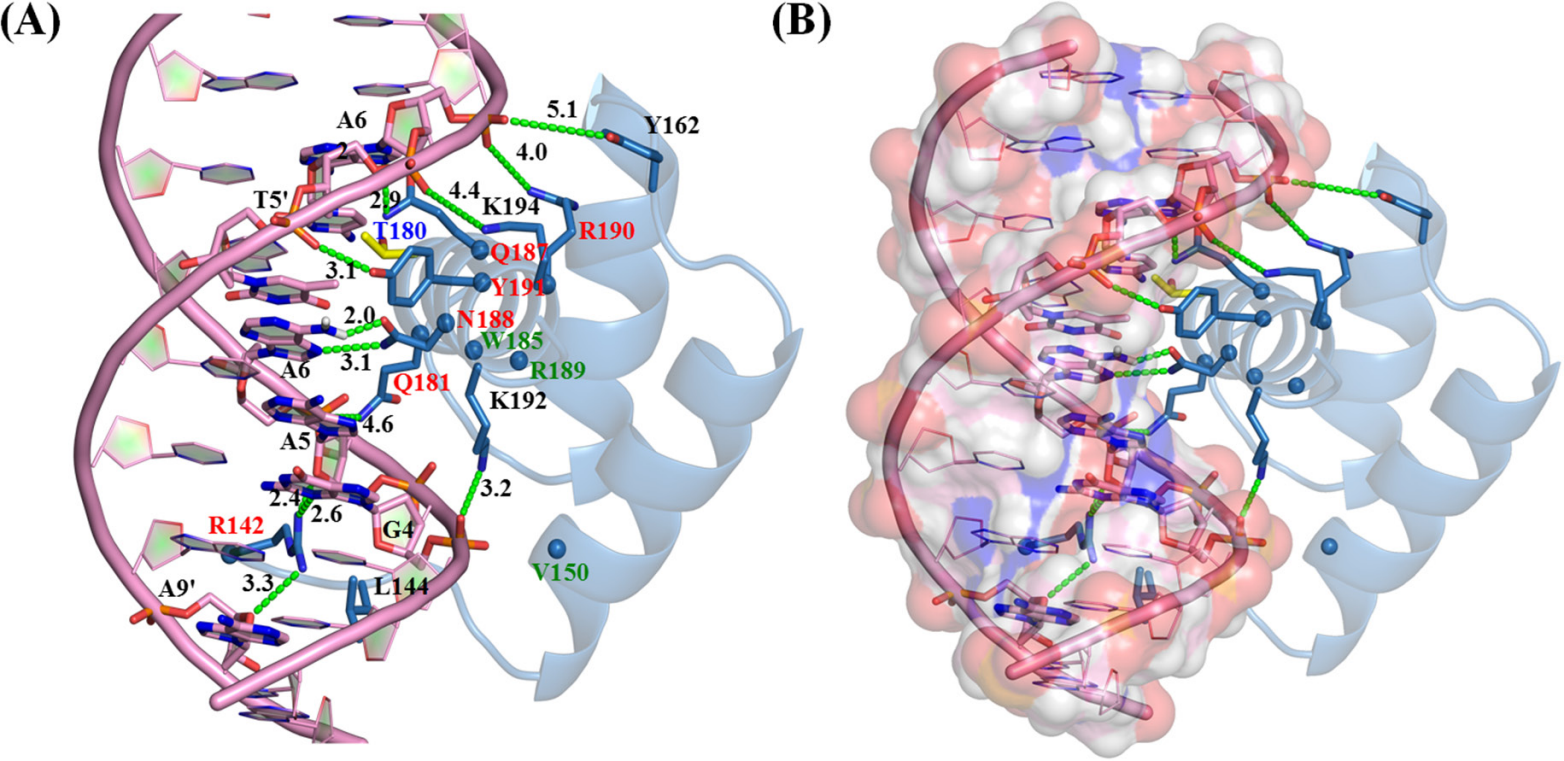
NKX2.5-DNA Interaction map: Residue level interaction between NKX2.5 and DNA (ANF-242) **(A)** All the residues having interactions with DNA (around 5.0 Å) are written in four categories: 1) only interacting residues are in black, 2) only mutations are in green color, 3) interacting and mutation both residues are red color and, 4) interacting and PTM residue in blue color. **(B)** DNA and amino acids are rendered as electrostatic surface view and licorice, respectively.

The interactions were achieved by protein-DNA docking via NP-dock (http://genesilico.pl/NPDock) and HADDOCK (https://haddock.science.uu.nl) tools (Figure 5 and S2A). From docking results, we found that the residues 141-197 of HD region are mainly participating in interaction with the DNA. The HD residues established the stable hydrogen bonds interacting with DNA are shown in Figure 5 and Supplementary Table 2. The protein-DNA complex was gained additional binding affinity through hydrophobic contacts with residues L144, F145, and I184 and the flexible polar contacts are found with residues R139, P145, R161, R168, I184, W185, and K192. The structural stability of the models was assessed by calculating the evolution time of potential energy and, the root-mean square deviation (RMSD) between *NKX2.5* (apo) and NKX2.5-DNA. From the MD simulation we observed that in terms of RMSD values, the NKX2.5-DNA complex is more stable, in which the mean RMSD value (11 Å) is lower than *NKX2.5* apo-form (14.5 Å) (Figure 4C), *NKX2.5* moves into more stable conformation upon binding of the DNA. Furthermore, we measure the time evolution of distance between C-alpha atom of D16@NKX2.5 and center of mass of nucleotide residue 3 to 7 of chain C and 16 to 19 of chain D of DNA. The mean average distance is ∼27 Å and the minimum distance throughout trajectory is 23 Å which is quite high to impact any significant effect of D16N on positioning of DNA (Supplementary Figure 2A, and Supplementary Table 2).

### Protein-protein docking: *NKX2.5* and *GATA4* binds at two binding regions (BR1 and BR2)

**Figure 6:**
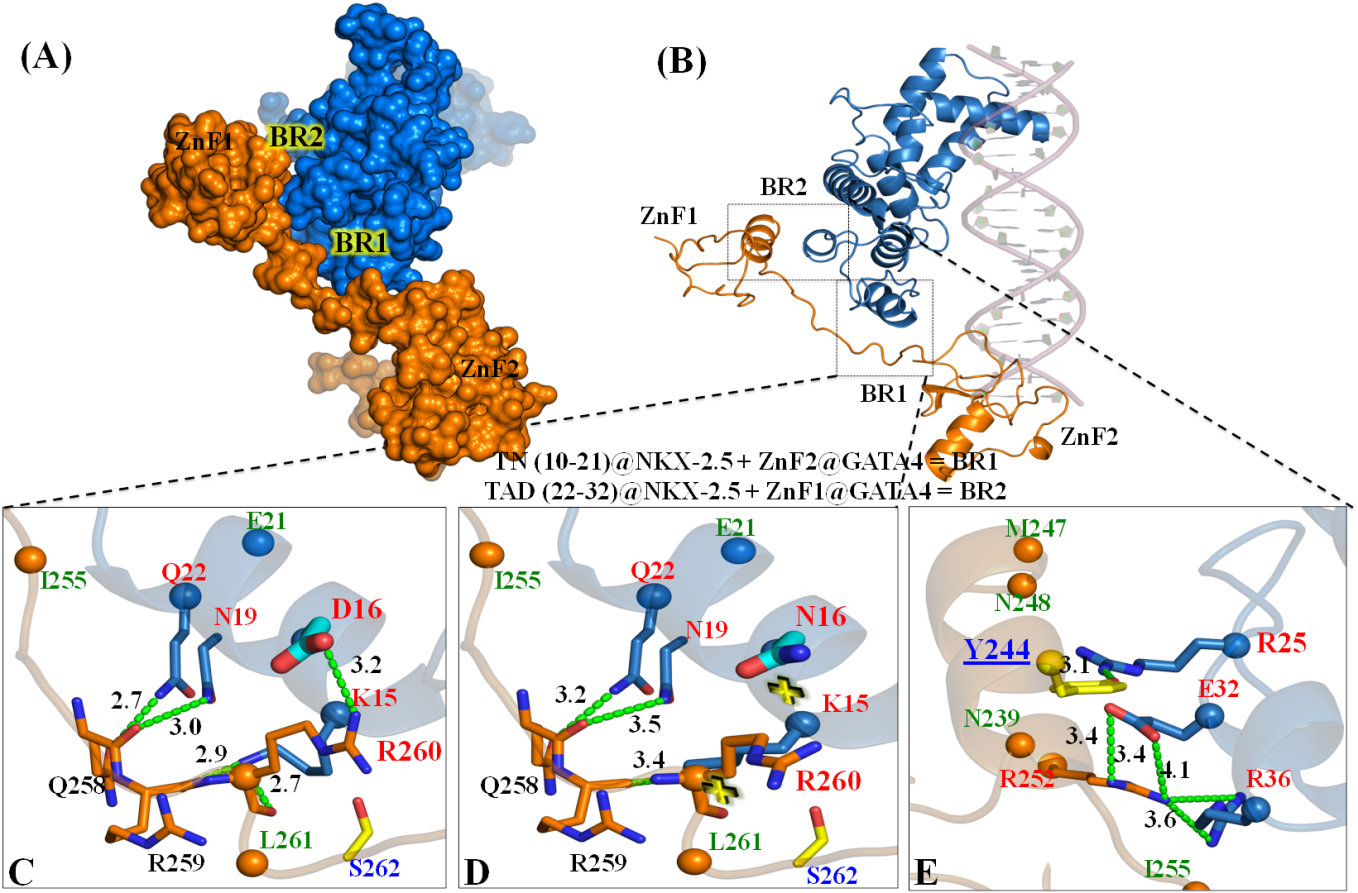
NKX2.5 −GATA4 interaction map: **(A)** In the interactions zones (BR1 and BR2) of *NKX2.5* (in blue surface) and *GATA4* (in orange surface) are shown. The binding region constituted by TN (10-21)@*NKX2.5* + ZnF2@*GATA4* = BR1, TAD (22-32)@*NKX2.5* + ZnF1@*GATA4* = BR2. **(B)** The *NKX2.5* and *GATA4* interaction shown in carton and rendered with same color. The DNA bound with *NKX2.5* is shown in dull pink color. The two binding regions (BRs), BR1 and BR2 are highlighted by dotted squares. The zoom-in view, of BR1 is shown in panel **(C)** and **(D)**, and BR2 is shown in panel **(E)**. In panels (C), (D), and (E), all the key residues are shown as stick and rendered as atom-wise (C: cyan, O: red, N: blue, S: yellow). The residues are categorized in four colors. Red: interacting residues with mutations, Green: only mutation, Black: only interacting and Blue: interacting and PTM. The novel mutation residue D16 (wild-type: in panel C) and N16 (mutant: in panel D) are shown in thick cyan color. (E) The BR2 interaction-map. Zn atom has been omitted from the Figure for the clarity (for Zn atoms see supplementary image S13).

The average structures of *NKX2.5* and *GATA4* extracted from last 80 ns of MD simulation were used for protein-protein (PP) docking, respectively. The docked-poses having docking energy above −30 kcal/mol were selected for best-pose analysis. The top 20 complex poses of *NKX2.5* and *GATA4*, the lowest energy poses (lowest docking energy and maximum number of conformers) was selected for molecular dynamics simulation of complex (Figure 4A and 4B). The interactions achieved after performing the rigid and flexible PP docking shows that amino acid residue 13-38 of N-term of *NKX2.5* are interacting with residue 238-263 of *GATA4* (Figure 6A and 6B and Supplementary Table 3). Prior to MD, the identified interface sites of the complexes were cross-checked by performing the binding site detection method through Schrodinger [36] (Supplementary Figure 7, and Supplementary Table 4). The site detection method identified the BR1 and BR2 as the most prominent interface site, which was corroborated well with the docking outcomes (Supplementary Figure 7). The site score and the volume of the cavity criteria were counted for the selection of the top sites [37].

### Molecular dynamics simulation study

#### RMSD

We build the models of NKX2.5+DNA with *GATA4* and *TBX5*. We find that in partner proteins *GATA4* is in the vicinity of D16N, however, *TBX5* is far away (Supplementary Figure 8). Therefore, the complex of *NKX2.5* and *GATA4* was selected for further analysis. The MD simulation was performed for each system: NKX2.5 (apo), NKX2.5-DNA, NKX2.5-GATA4, NKX2.5-DNA-GATA4 (WT) and NKX2.5-DNA-GATA4 (MT) (Figure 4C). The system like NKX2.5-DNA (mean RMSD: 11 Å) and NKX2.5-GATA4 (mean RMSD: 10 Å) alone is not dynamically stable, since both have high RMSD (≥10 Å), as in case of NKX2.5-DNA the TN domain is free to move, however, in case of NKX2.5-GATA4 the HD domain reflects high flexibility (Figure 4C and 4D). From the RMSD analysis, we find the NKX2.5-DNA-GATA4 (WT) is the most stable, in which mean RMSD (2.5 Å) is far below than other sets of complexes (Figure 4C). This behavior is clearly reflected in the probability distribution of RMSD of MD systems (Figure 4C) and indicates system NKX2.5 (D16)-DNA-GATA4 (WT) as a true and complete system for the dynamic study of mutation (Figure 4B). The stable WT system is used to check the mutational effect. In WT system the residue D16@NKX2.5 is changed into N16@NKX2.5 to make mutated system, NKX2.5 (N16)-DNA-GATA4 (MT). From the RMSD analysis between system WT and MT, we find a significant ∼4.0 Å difference, indicating that the MT system (mean RMSD: 6.5 Å) dynamics is different than the WT system and a huge decrease in stability occur after D16N mutation.

#### Domain movement (distance HD-TN)

Since the *NKX2.5* domains (TN+TAD+HD) are essential to hold the DNA (from HD side) and to have interaction with *GATA4* (from TN side), as reflected from the dynamics of system NKX2.5-DNA and NKX2.5-GATA4, domain movement graph Figure 4D. Therefore, the movement between HD and TN is essential to understand the dynamic pattern of the WT and MT systems. From the complex assembly obtained from MD simulation (average structures), the TN domain of *NKX2.5* is stably associated with *GATA4*. Since the mutation D16N, localized in the same TN domain, therefore, to observe the changes in the dynamics between WT and MT, the center of moss of HD and TN domain was picked for domain movement analysis along the whole trajectories. There were no considerable differences were observed in domain movement of WT and MT complexes (Figure 4D).

#### Distance analysis at BR1 and BR2 in WT and MT

Furthermore, to see the effect of D16N mutation, we performed the distance analysis of BR1 and BR2 sites by taking the center of mass of interaction area (Figure 4B&4F). We observed a significant variation in site BR1 in MT system than the WT system, which pronounced very clearly in the population distribution. In WT the trajectory is distributed with mean value of 5.3 Å, while in mutant it is bimodal and also distributed with mean value of 9Å, indicating a remarkable difference at BR1 site (Figure 4F). However, similar to domain movement of HD-TN, the distance at BR2 site is also stable. It indicates that BR1 site severely affected by D16N. The Root mean square fluctuation (RMSF), which measures of the average fluctuation of the residue over time, was also performed to have a clear picture of dynamic changes at BR sites. The RMSF of the C_ɑ_ atom of the entire residue over different time frame was plotted to measure the dynamic changes that occur at the level of single residues and analyses the fluctuation of the interface residue of *NKX2.5* over the simulation time. The RMSF difference plot was also generated by plotting the difference in RMSF of WT and MT; this was done to determine the regions that have shown the significant structural changes during the MD simulation. The RMSF values clearly indicate that MT has shown significant changes at BR1 compared to the BR2 sites (Figure 4E). The residues that have large fluctuation in MT are 1-25 (BR1), 35-45 (BR2), 79-81, 85-87, 102-108 and 134-144. As compared to the BR2 site, the fluctuation at BR1 is very high and this is well represented by the distance time evolution graph also (Figure 4F). The distance time evolution between Cɑ atom of residue 16 of *NKX2.5* and R260@GATA4 was plotted for system WT and MT (Figure 4G). In case of system WT, the distance is consistent throughout the trajectory with unimodal distribution in probability plot and mean average distance 5.3 Å. While, in case of the MT system, distance after 40ns jump to 14 Å and distribution plot is trimodal.

#### Interaction analysis

The MD trajectory shows that the complex is stable, and the averaged structure was extracted from the last 80 ns simulation to generate the stable interaction map between *NKX2.5* and *GATA4*. The interface site was quantified in terms of residue-wise interaction energy analysis and, the life-time occupancy of HBs (Figure 4H and 4I). The interaction pattern at the interface site of the complex was quantified in terms of HBs and HpH contacts. The HBs formed at BR1 (ZnF2) are *NKX2.5*@K15:*GATA4*@R260, *NKX2.5*@K15:*GATA4*@R259, *NKX2.5*@D16:*GATA4*@R260, *NKX2.5*@N19:*GATA4*@Q258 and *NKX2.5*@Q22:*GATA4*@Q258 (Figure 6C and 6D). At BR2 (ZnF1) region, the HBs formed are *NKX2.5*@R25:*GATA4*@Y244, and *NKX2.5*@Q32:*GATA4*@R252 (Figure 6E). Additionally, the residues strengthen the binding affinity between *NKX2.5* (residues V14, I17, L18, L20, L27, A28, A29, A30, L33, A35 and L37) and *GATA4* (residues A263, L261, P257, I255, L254, P253, I250, M247, L243, C241 and C238) by establishing the hydrophobic contacts. The HBs that persist longer time are also showing the higher interaction energy (Figure 4H and 4I), however, the common interaction-life and interaction-energy was found in residues K15, D16, and R25 of *NKX2.5* (Figure 4H and 4I).

### Reported pathogenic mutations

Since we identified a novel pathogenic missense mutation and, from the residue-wise analysis we confirmed that D16 contribute significantly and it involved in the active association of *NKX2.5* and *GATA4* which is essential for their biological function. This provides a clue that those residues that are actively involved in the association of *NKX2.5* and *GATA4*, possibly are the pathogenic mutants. Therefore, the literature was explored with the aim to identify the other pathogenic mutations in *NKX2.5* and *GATA4*.

#### NKX2.5 HD regions

We found 18 point-mutations from 15 residues of HD. [10, 11, 38] These mutations are R142, Q149, V150, Q160, R161, Q170, L171, T178, S179, Q181, W185, Q187, N188, R190 and Y191 (Figure 5 and Supplementary Table 5).From the residue wise analysis, we noticed that among these reported pathogenic mutations, most of them are those which are involved in making hydrogen bonds (HBs) with DNA like R142, R161, Q181, W185, N188, R190 and Y191, which underscores the functional importance of this region. Also, we observed that the mutations in amino acids participating in key polar interaction (PI) are more prone to develop into disease state. All the residues having interactions with DNA are analyzed in four categories: (a) only interacting residues either HBs or hydrophobic (HpH) (4/14), (b) only reported pathogenic mutations (3/14), (c) interacting and pathogenic both (6/14), and (d) interacting and PTM (1/14). Our docking outcomes corroborate nicely with the previously published crystal data [6]. From the mutation list of HD region and the interaction pattern between DNA and HD, we found that most of the residues are common, indicating that it might be possible that the loss of polar contacts would contribute significantly in the diseased causing state.

#### Pathogenic mutations in *NKX2.5* and ZnF domain of *GATA4*

Similar to HD region, the second hub of the mutations is localized around BR1 and BR2 interface site of *NKX2.5* and *GATA4*. The BR1 site constitutes 3 mutations having interaction with *GATA4* residues; while at BR2 site, the total number of 6 mutations is reported. Interestingly, out of 6 mutant residues, 4 same residues are involved either in terms of HBs or HpH contacts. (Table 4) Most of the interactions localized at BR sites are transient, indicating that these two proteins come together for a short time as both of them constitute of disordered zone which transformed into ordered form during interactions. Since occurrence of mutations is the well-known cause of disease, therefore, we tried to map all the possible reported mutations in NKX2.5-GATA4 interface (Table 4). Interestingly, most of the known diseases causing mutations are those residues, which we found to contribute significantly in the stability of NKX2.5-GATA4 complex through residue wise analysis. Residue D16@*NKX2.5* and R25@*NKX2.5* contribute most significantly in stability of NKX2.5-GATA4 complex (Figure 4H and 4I).

**Table 4:** Amino acid residues of *NKX2.5* and *GATA4* present in cutoff value 8.0 Å (cut off taken through pymol)

MOTIFs	*NKX2.5*				*GATA4*			
	Residues	PI	MT	PTM	Residues	PI	MT	PTM
**Binding Region 2(BR2)**					I255			
	V14				K256			
	K15	K15	K 15 I		P257			
	**D16**	**D16**	**D 16 N**		Q258	Q258		
	I17				R259	R259		
	L18				R260	R260	R260Q	
	N19	N19	N19S		L261		L261P	
	L20				S262			S262
	E21		E 21 Q		A263			
	Q22	Q22	Q 22 K/P/R		S264			S264
	Q23				R265			
	Q24				R265		R 265 TER^*^	
**Binding Region 1(BR1)**	R25	R25	R 25 C		N239		N239D	
	S26				A240			
	L27				C241			
	A28				G242			
	A29				L243			
	A30				Y244	Y244	Y244C	Y244
	G31				H245			
	E32	E32	E 32 K		K246			
	L33				M247		M247T	
	S34				N248		N248S	
	A35				G249			
	R36	R36	R 36 S		I250			
	L37				N251			
	E38				R252	R252	R252P	
	A39				P253			
	T40				L254			
					I255		I255T	

Residues participated in direct polar interaction, mutations, and PTM localize in the interaction zone are listed. PI = Polar Interactions, MT = Mutations. Bold amino acids are those, which are identified by this work.

### Computational mutagenesis and alanine scanning

**Figure 7:**
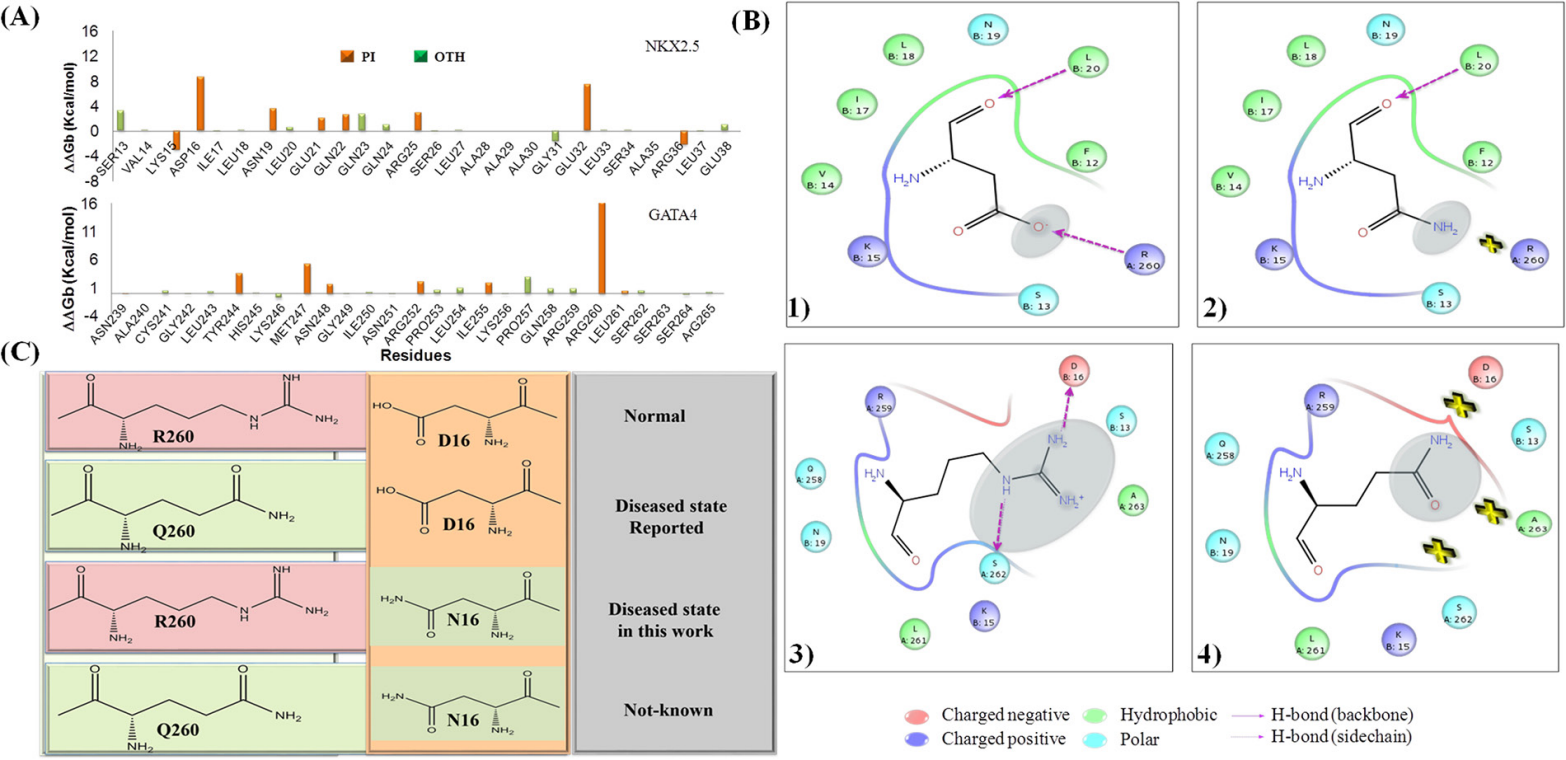
**(A)** Alanine Scanning: Results summary for BioLuminate alanine scanning of interface residues of NKX2.5-GATA4 protein complex and change in binding free energy/affinity (ΔΔG_b_) (kcal/mol). Residue participated in the polar interaction (PI), shown as red bar and rest all other interface residue (OTH), shown as green bar. ΔΔG_b_value higher than zero means decrease in binding affinity and value less than zero means increase in binding affinity. Upper panel belongs to *NKX2.5*, and lower panel belongs to *GATA4*. **(B)** 2D interaction map of D16: 2D map represent the changes in interaction pattern of D16/N16 and R260/Q260 in wild type and Mutant form at BR1 site. (1 and 2) Interaction-map, within 5 Å from *NKX2.5* residue: 1) D16; 2) N16. (3 and 4) Interaction-map, within 5 Å from *GATA4* residue: 3) R260; 4) Q260. **(C)** Schematic representation: Key residue interactions of D16N@*NKX2.5*:R260Q@*GATA4* and their physiological outcomes.

The affinity and specificity in protein interface act as a key to regulate/modulate protein-protein interaction. It is a well-known fact that formation of protein-protein complex/s depends on few interface residues contributing most in the binding free energy, called as ‘*hotspot*’ [39]. Alanine scanning is the powerful tool to find out binding *hotspot* at the interface of protein-protein complex. It measures the net change in the binding free energy (ΔΔG_b_) of a protein-protein complex upon mutation of amino acid residue to alanine. We have scanned all interface residue with Schrodinger module BioLuminate [40], and two other web servers DrugScorePPI [39] and BeatMusic [41]. We performed alanine scanning of all interface residues in both *GATA4* and *NKX2.5* to find *hotspot* residues. We found that in all the cases Bioluminate, DrugScorePPI and BeatMusic, the residues, which are forming direct polar interactions, after mutation in alanine, lead to significant decrease in the binding affinity compared to other residues, which are not participating as a polar interaction. Bioluminate identified D16@*NKX2.5* (ΔΔG_b_, 8.65 kcal/mol) as a key residue and have second most decreased binding affinity after R260@GATA4 (ΔΔG_b_, 16.32 kcal/mol) in alanine scanning of interface residues of both *NKX2.5* and *GATA4* (Figure 7A, and Table 5 and 6). This outcome is well supported by DrugScorePPI (0.8 kcal/mol, top_score 1.37 kcal/mol) and BeatMusic (0.9 kcal/mol, top_score 1.29kcal/mol) (Table 5 and 6). The salt-bridges were identified by DrugScorePPI between D16@*NKX2.5* and R260@*GATA4* at BR1 and, E32@*NKX2.5* and R252@*GATA4* at BR2. It was reported that the residues involved in salt-bridge formation are considered as key *hotspot* residue. Furthermore, we also performed the mutagenic analysis for reported mutants at interface through BioLuminate and BeatMusic. The mutagenic analysis corroborates well as it follows the same trend of change of binding free energy as we found in the case of alanine scanning. This analysis confirmed a significant drop of decrease in binding energy especially in residues D16, E32 of *NKX2.5* and Y244 and R260 of *GATA4* (Supplementary Table 6).

**Table 5:** Results summary for alanine scanning of interface residues of *NKX2.5* of NKX2.5-GATA4 protein complex

Residues	Mutation to Alanine	Change in binding affinity (∆∆G_b_ kcal/mol)			Possible Salt-bridges (DrugScorePPI)
		BioLuminate	BeatMusic	DrugScorePPI	
Ser13	K → A	3.31	0.92	0.46	-
Val14	V → A	0.08	0.03	0.06	-
Lys15	K → A	-3.07	0.92	1.13	-
**Asp16**	**D → A**	**8.65**	**0.68**	**0.8**	**X**
Ile17	I → A	-0.03	0.08	-	-
Leu18	L → A	0.12	0.16	0.16	-
**Asn19**	N → A	**3.59**	**1.26**	**1.37**	-
Leu20	L → A	0.63	0.08	0.08	-
Glu21	E → A	2.09	0.04	-	-
Gln22	Q → A	2.58	0.43	0.04	-
Gln23	Q → A	2.78	0.28	0.54	-
Gln24	Q → A	1.04	0.3	-	-
**Arg25**	R → A	**2.95**	**0.44**	**0.34**	-
Ser26	S → A	-0.01	-0.24	-	-
Leu27	L → A	0.02	0.3	-	-
Ala28	-	-	-	-	-
Ala29	-	-	-	-	-
Ala30	-	-	-	-	-
Gly31	G → A	-1.74	0.45	-	-
**Glu32**	E → A	7.44	0.99	1.2	X
Leu33	L → A	0.02	0.6	0.06	-
Ser34	S → A	0.06	0.05	-	-
Ala35	-	-	-	-	-
Arg36	R → A	-2.26	0.57	0.35	-
Leu37	L → A	-0.08	0.2	-	-
Glu38	E → A	1.02	-0.09	-	-

The key residues are highlighted. ∆∆G_b_ value higher than zero means decrease in binding affinity and value less than zero means increase in binding affinity.

**Table 6:** Results summary for alanine scanning of interface residues of *GATA4* of NKX2.5-GATA4 protein complex

Residues	Mutation to Alanine	Change in binding affinity (ΔΔG_b_ kcal/mol)			Possible Salt-bridges (DrugScorePPI)
		BioLuminate	BeatMusic	DrugScorePPI	
Asn239	N → A	0.1	-0.03	-0.06	-
Ala240	-	-	-	-	-
Cys241	C → A	0.52	0.34	0.28	-
Gly242	G → A	-0.04	0.04	-	-
Leu243	L → A	0.29	0.18	0.27	-
**Tyr244**	**Y → A**	**3.61**	**1.72**	**0.55**	-
His245	H → A	0.12	0.20	-	-
Lys246	K → A	-0.78	0.11	-	-
Met247	M → A	**5.28**	**0.51**	**0.19**	-
Asn248	N → A	1.79	0.43	0.32	-
Gly249	G → A	-0.02	-0.13	-	-
Ile250	I → A	0.18	0.58	0.67	-
Asn251	N → A	-0.09	0.23	0.07	-
**Arg252**	R → A	**2.18**	**1.41**	0.45	X
Pro253	P → A	0.62	0.32	-	-
Leu254	L → A	0.98	0.16	0.16	-
Ile255	I → A	1.98	0.52	0.52	-
Lys256	K → A	-0.13	0.00	0.05	-
Pro257	P → A	2.85	0.27	-	-
Gln258	Q → A	0.82	0.63	0.37	-
Arg259	R → A	0.86	0.15	0.01	-
**Arg260**	**R → A**	**16.32**	**1.77**	**1.70**	**X**
Leu261	L → A	0.53	-0.06	0.07	-
Ser262	S → A	0.47	0.46	0.26	-
Ser263	S → A	0.34	0.46	-	-
Ser264	S → A	-0.24	0.25	-	-
Arg265	R → A	0.22	0.19	-	-

The key residues are highlighted. ∆∆G_b_ value higher than zero means decrease in binding affinity and value less than zero means increase in binding affinity.

Furthermore, we also performed the Post Translational Modifications (PTMs) identification, as it is known that PTM can induce conformational changes [24, 42], and we find three phosphorylation sites at ZnF region of *GATA4*: S262, and S264 at BR2 and Y244 at BR1 sites (Figure 6) [43, 44].

## DISCUSSION

In the present study, we have looked *NKX2.5* mutations among 100 CHD patients having ASD, VSD, TOF and SV. Among several reported mutations, we found one novel missense mutation D16N in CHD patients and associated with a VSD phenotype. This D16N mutation is present in the TN domain of *NKX2.5* and conserved in human, cattle, monkey, pig, dog, rat and mouse. (Figure 1B) To know the functional significance of this missense mutation, we performed bioinformatics analysis using PMut, PROVEAN, SIFT, PANTHER, PHD-SNP, SANP, Polyphen-2 and PredictSNP webserver. PMut, SIFT, PROVEAN, PANTHER, PHD-SNP, SANP, and PredictSNP strongly indicated this mutation is pathogenic. Indeed, PolyPhen-2 act as outlier as it recognizes this D16N mutation as not pathogenic, however, PolyPhen-2 which included in PredictSNP pipeline shows this mutation as pathogenic. The mutation prediction tools corroborate nicely with our clinical outcome, motivate us to do further in-depth structural analysis to find localization, molecular mechanism, and its association in perturbed biological function.

**Figure 8:**
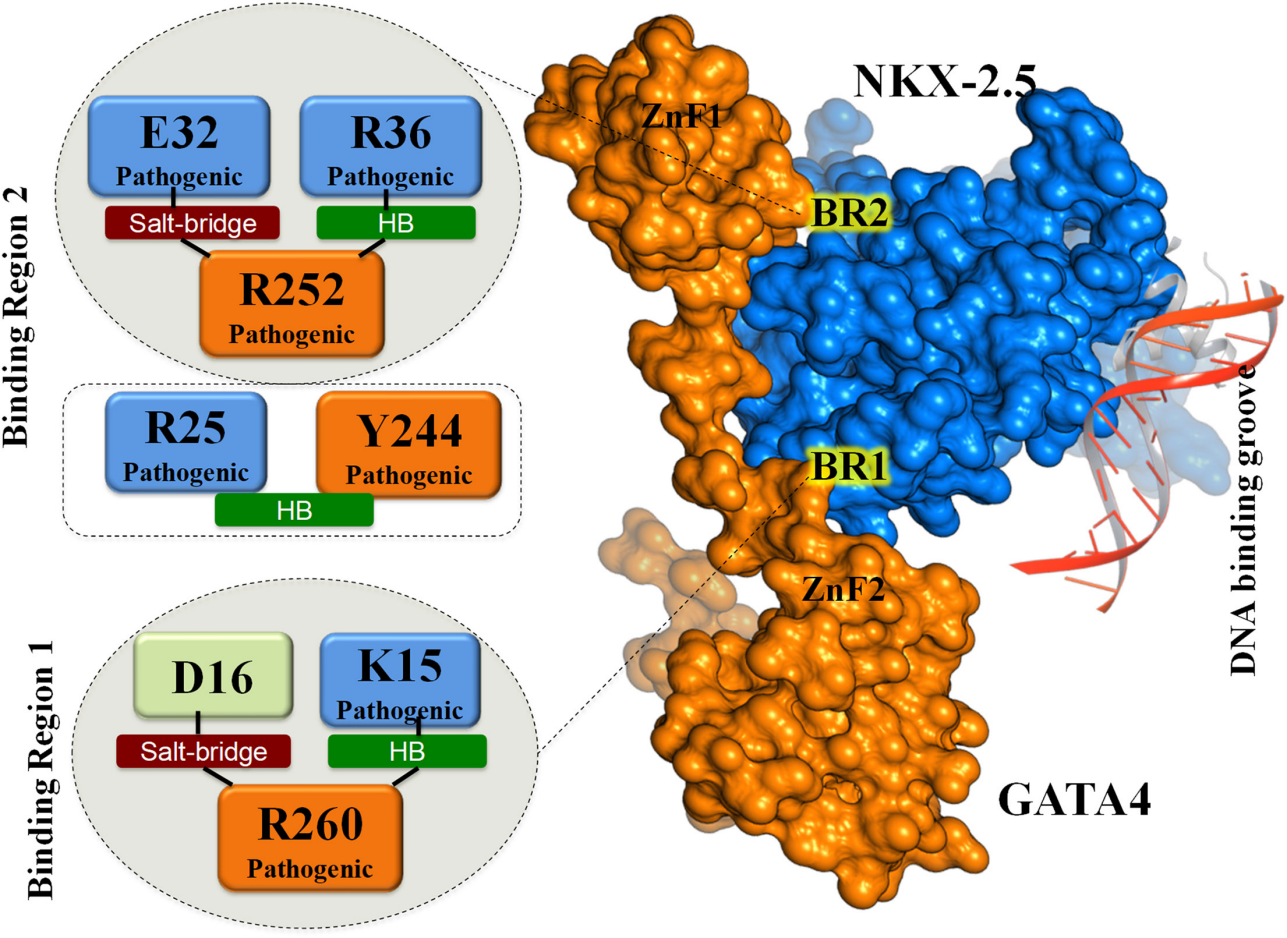
Biological significance of identified key residues: The key interaction triads (in form of salt-bridges and HBs) are highlighted at identified site of BR2 (ZnF1) and BR1 (ZnF2) Except residue D16 (in lime color), all other residues of *NKX2.5* (in blue) and *GATA4* (in orange) are reported as “pathogenic mutations”.

The structural and biochemical properties of the disordered regions are ideal for TFs as they can mediate specific recognition of interaction partners or co-ordinate regulatory events in space and time. [45–47] The function of intrinsically disordered proteins may be controlled by some factors like mutations, endo/exogenous ligands and/or post-translational modifications that lead to structural changes. Previously, no functional role was defined for TN domain in *NKX2.5*. In this study, we found that amino acid residue D16 present in the TN domain and playing a crucial role to interact with *GATA4*. From the protein-protein interaction studies, we identified two *hot-spots* i.e BR1 and BR2. The identification of *hot-spots* depends on three criteria: a. key interactions, b. reported mutations and, c. localization of PTM sites. Interestingly, we noticed that most of the polar interactions are also reported mutations (Table 4 and S5). It is observed that the localization of D16, and its nearby regions are the hub of CHD mutations in *GATA4* and *NKX2.5.* Additionally, we also identified the phosphorylation site from the database K52@*NKX2.5*, S262@*GATA4*, S264@*GATA4*, and Y244@*GATA4* in the vicinity of the interaction sites. Therefore, the key interaction pattern, localization of pathogenic mutations and presence of PTMs at interface site in both proteins, all together justified the identification of two *hot-spot* BR1 and BR2. The most stable interactions are *NKX2.5*@D16:*GATA4*@R260 at BR1 site and *NKX2.5*@R25:*GATA4*@Y244 at BR2 site. The residue R260Q@*GATA4* is reported as a pathogenic mutation, causing ASD and VSD. [10] Interestingly, *NKX2.5*@K15 and our identified mutation *NKX2.5*@D16 form a stable hydrogen bond with *GATA4*@R260. The loss of polar contacts with variation at residue R260Q might induce the conformational change and destroy the structural-zip between *NKX2.5* and *GATA4* at BR1 site and, could be a reason of pathogenicity (Figure 7B and 7C). The similar affect possibly reflects if genetic variation occurs at residue D16 (Figure 7B and 7C), indicating the importance of this residue towards pathogenicity. The D16 mutation might be responsible for pathogenicity can be further claimed, by the interaction pattern and biological significance of the residues participating in interaction at BR1 and BR2 site. We found, at the BR2 site, a set of three residues (one acidic and two basic) with one salt-bridge (E32@*NKX2.5*:R252@*GATA4*) and one hydrogen bond (R36@*NKX2.5*:R252@*GATA4*) make an interaction triad. The similar interaction triad pattern, with one salt-bridge (D16@*NKX2.5*:R260@*GATA4*) and one hydrogen bond (K15@*NKX2.5*:R260@*GATA4*), among a set of similar type of three residues (one acidic and two basic) is also formed at the BR1 site. The amino acid variations in the residues of interaction triads at both sites are reported as pathogenic [10] except D16 which is reported as pathogenic in this work (Figure 8). Additionally, one more hydrogen bond R25@NKX2.5:Y244@GATA4 is identified at the BR2 site and, the amino acid mutations R25C@*NKX2.5* and Y244C@*GATA4* are reported as a pathogenic diseased state [10]. From the structural modeling data, we found that these interaction patterns would be lost due to variation at any of these amino acids. Thus the mutation/s at these amino acids possibly disturbs the interface interaction map which leads to the unstability of the complex.

Other residues of *NKX2.5* (N19 and Q22) and *GATA4* (Q258 and R259) are also contributing in zipping the *NKX2.5-GATA4* interactions at BR1 and BR2 sites by making hydrogen bonds. In general, these two TFs are mostly unstructured disordered form. For regulation of transcription, these two TFs come close together at some binding sites and the binding regions are transformed from unstructured-to-structured form. It seems that the hydrogen bonds between K15, D16, N19, Q21 and R25 of *NKX2.5* and Y244, Q258, R259 and R260 of *GATA4* at BR1 and BR2 sites are acting like a “zip” to hold the interactions at these two sites. The regions of *GATA4* which interact with D16@*NKX2.5* are unstructured, flexible and have two reported phosphorylation sites i.e. S262 and S264. The phosphorylation at these sites might induce the conformational change and possibly play a role in on/off switching mechanism pattern during interaction between NKX2.5-GATA4. The phosphorylation sites are known to induce some conformational changes and helping to regulate transient interactions specially at interfaces and binding *hot-spot*. [48, 49] Result of residue wise analysis and in-silico alanine scanning mutagenesis have given clue that the interface site BR1 and BR2 act as binding hot spot, and residue D16@NKX2.5 act as a key hot spot residue. Interestingly, except D16, all other residues are reported as pathogenic mutations indicating the importance of BR1 and BR2 sites for transcription regulation (Figure 6 and 7). The genetic variations of BR sites residues have already been documented to perturb the transcriptional regulation. We have noticed that D16 residue directly perturb the hydrogen bond with R260 of *GATA4* and parallel hindering other key interactions too. These alterations at residue level induce significant structural conformation-orientation changes and loss of secondary structures, by which it unzips the binding of *NKX2.5* and *GATA4* at BR sites and induces perturbed transcription regulation. Therefore, we concluded that D16N mutation of *NKX2.5* might be pathogenic as the variation in this residue, not only become a cause of losing the hydrogen bond with R260 of *GATA4*, also perturbing the interaction pattern at BR1 and BR2 sites significantly. The perturbation of this interaction may impair the downstream pathways crucial for heart development. It is reported that protein *NKX2.5*, has an autoinhibitory domain and, alone does not have significant effects on the expression of downstream target genes such as A1 adenosine receptor, α-cardiac actin, cardiac troponin T, cardiac ankyrin repeat protein, leucine-rich repeat containing 10 and inhibitor of differentiation/DNA binding-2. Direct physical-interaction of *NKX2.5* with *GATA4* is crucial to regulate the expression of the above essential cardiac genes in a synergistic manner [5, 50, 51]. It is well established that *NKX2.5* cooperatively with *GATA4* facilitates its activating and repressing functions. The interaction between *NKX2.5* and *GATA4* might also be important for the function as a repressor of ion channels and its downstream target genes. Therefore, We hypothesized that the mutation D16N possibly destroy or lose the interaction between *NKX2.5* and *GATA4* and thus may altered the transcription regulation and eventually leads to loss of several activator and repressor function of this complex.

In summary, we have identified a novel *NKX2.5* genetic variation, D16N which is associated with disease VSD in South Indian patients. This work underscores the importance of interaction sites which is the hub of pathogenic mutations. We have identified some key residues from *NKX2.5* (Y162, K192, and K194) and *GATA4* (Q258 and R259), which contributes substantially in *NKX2.5* and *GATA4* complex assembly. We have also found two phosphorylation (S262, and S264) sites in the vicinity of the interaction site that might be responsible for conformational changes, as a prerequisite for TFs regulation. The genetic variations at these amino acids possibly alter the function of the proteins. To further confirm, there is a need to make a genetic mouse model with *NKX2.5* D16 mutation and development of CHD phenotype. Overall, this work highlights a novel pathogenic mutation D16N in *NKX2.5* causing VSD and, underscores the structural mechanistic as how D16N can induce the structural-functional divergence that possibly leads to the disease state.

## MATERIALS AND METHODS

### Clinical evaluation and sample collection

In this study, we used two different selection criteria during collection of sample. The first criteria were CHD types such (ASD, VSD, TOF and SV). The numbers of CHD patient diagnosed in south part of India are much more in these four categories. The second criteria were demographics i.e. selection of the study population made according to geographical location, speaking the Dravidian language (Dravidians) and living in southern India. By considering both selection approaches we collected samples of CHD patients and control group. All the CHD patients studied (n=100) were outpatients and were recruited at the Innova Children’s Heart Hospital in Hyderabad, India. Control samples (n= 200), who belonged to the same ethnicity, were collected at Innova Children’s Heart Hospital and other hospitals in Hyderabad. Patients were clinically evaluated by pre-designed protocol that includes 2D echocardiography, color doppler and ECG. Atrial septal defect (ASD), ventricular septal defect (VSD), tetralogy of fallot (TOF), and single ventricle (SV) were considered for the present investigation. Informed written consent was received from all CHD patients and control samples. The study was approved by the research advisory committee and institutional ethical committee of Innova Children’s Heart Hospital (IEC/IRB No. 001/2010), Hyderabad, India.

### Genotyping

DNA was isolated from blood samples according to the protocol of Sambrook et al., 1989. [52] *NKX2.5* sequence from the ENSEMBL (ID: ENSG00000183072; www.ensembl.org) was used to design primer employing primer 3 software (http://frodo.wi.mit.edu/) and synthesized commercially (Eurofins, India). We investigated the genomic DNA of CHD patients for variations in the entire coding and untranslated regions (3’ UTR and 5’ UTR) of *NKX2.5* gene. Detailed sequences of all primers used in this study have been summarized in Supplementary Table 7. We performed PCR, sequencing and details methods followed according to mattapally et al, 2015. [19]

### Mutation analysis

The raw sequence data were analyzed and carefully edited using the sequence analysis software. The edited sequences were assembled with reference sequence using DNA Star and Autoassembler software (Applied Biosystems, USA). All the variant sites, compared to the reference sequence were noted down. Genetic Association, Hardy–Weinberg equilibrium and Chi-square test were computed by using Plink software. Multiple alignments of *NKX2.5* amino acid sequences from different species across mammals were done by ClustalX [53]. Pathogenic potential of identified missense mutations from CHD patients was predicted by different webservers such as SIFT, PMut, PROVEAN, PANTHER, PHD-SNP, SNAP, polyphen2 (http://genetics.bwh.harvard.edu/pph2/), and PredictSNP.

### Statistical analysis

Statistical analysis was performed with Plink software and level of significance was set to a p-value of 0.05. The significance of deviations from Hardy-Weinberg equilibrium was tested using Plink software, we used cut off p-value 0.05; and for association analysis, we used same cut off p-value (0.05). We did association analysis and generated pictures with R basic packages (R version 3.0.2, 2013).

### Molecular modeling study

To conduct the 3D model of *NKX2.5*, the BLAST (from NCBI) was performed with the aim to identify the existing crystal structures with high identity and similarity. Additionally, we performed disorder tendency and protein binding analysis through DISOPRED, which highlight the probability estimation of each residue in the sequence. All details of the molecular modeling of the *NKX2.5* model (N-term + HD) and *GATA4* model were given in the material and method section in supplementary file. We have also described the method part of, finding of NKE motif in ANF promoter and Preparation of DNA, protein-DNA docking (NKX2.5-DNA), protein-protein docking (P-P docking) of NKX2.5-GATA4 complex in details in the material and method section in supplementary file.

### Molecular dynamics simulation

Molecular dynamic (MD) study for all complexes was carried out using Desmond. [54] The molecular systems were first built using System Builder panel. The parameters were assigned from inbuilt OPLS3 force field. “TIP3P” water model [55] was used to solvate the systems using orthorhombic box with distance of 10Å from all sides of protein complex [56]. System was electrically neutralized by adding appropriate counter Na+/Cl- ions. The prepared systems were further minimized using steepest descent algorithm for 2000 iterations with convergence threshold of 1 kcal/mol/Å. The minimized systems were then equilibrated using default algorithm which included two stages of minimization (restrained and unrestrained) followed by four stages of MD runs with gradually diminishing restraints using NVT at 300K, NVT at 700K, NPT at 300 K, and NPT at 300K respectively. Finally, 50 ns of MD simulation was performed using NPT ensemble wherein Nose-Hoover Chain thermostat [57] was used for temperature coupling at 300 K and Martyna-Tobias-Klein barostat [58] for pressure coupling at 1 atm. Long range coulombic interactions were calculated using smooth Particle-Mesh-Ewald method [59] while a cut-off of 9.0 Å for short range electrostatics contributions. Coordinates and energy were recorded every 10 ps to yield 5000 frames.

### Computational mutagenesis and alanine scanning

Alanine scanning mutagenesis was carried out through Schrodinger BioLuminate tool and web servers, DrugScorePPI and BeatMusic. The PDB file of NKX2.5-GATA4 complex was prepared and submitted to DrugScorePPI and BeatMusic webserver and the chain of interest was selected for alanine scanning. The residues scanning in BioLuminate was performed through residue scanning/affininty maturation panel in biologics with refinement option set to side-chain prediction and backbone minimization, the cutoff distance was set to 0.0. BioLuminate, DrugScorePPI and BeatMusic all calculate the net change in binding affinity (ΔΔGb) produced by single point mutation. Schrodinger module BioLuminate utilizes MM-GBSA approach with force field OPLS2005 and solvent model VSGB to calculate ΔΔGb. DrugScorePPI, work on knowledge-based scoring function and automatically detects all interface residues within 5Å cutoff and mutate them one by one to alanine and calculate ΔΔG_b._ BeatMusic depends on the residue-based set of statistical potential obtained from known protein structure.

## SUPPLEMENTARY MATERIALS FIGURES AND TABLES


