# Supplementary material for: Computational modeling suggests impaired interactions between NKX2.5 and GATA4 in individuals carrying a novel pathogenic D16N NKX2.5 mutation
Source: Oncotarget. 2018 Feb 9;9(17):13713–32. doi: 10.18632/oncotarget.24459 (PMC5862610; doi:10.18632/oncotarget.24459)
Supplement: Supplementary file 1 [file oncotarget-09-13713-s001.pdf]

Computational modeling suggests impaired interactions between NKX2.5 and GATA4 in individuals carrying a novel pathogenic D16N NKX2.5 mutation

SUPPLEMENTARY MATERIALS

MOLECULAR MODELING

Modeling of the *NKX2.5*

Human *NKX2.5*, UniProt id. P52952 (http://www.uniprot.org) is 324 amino acids long in length. It is well classified into three regions N-terminal (N-term), homeodomain (HD), and C-terminal (C-term) (Figure 1C). HD localized at center (from residue 138-197 amino acid) and highly conserved. The N-term (from residues 1-137) contain, tin-man domain (TN) and transcriptional activation domain, and C-terminal (from residues 198-324), contain nucleotide kinase2 (NK2) domain (from residue -234aa) [1, 2].

To conduct the 3D model, the BLAST (from NCBI) was performed with the aim to identify the existing crystal structures with high identity and similarity. Overall, very less sequence similarity was found for N-term and C-term regions, however, exceptionally high similarity was found for HD region. Additionally, we performed disorder tendency and protein binding analysis through DISOPRED [3] and IUPRED [4], which highlight the probability estimation of each residue in the sequence (Figure 1E), which claimed well that except HD region, N-term and C-term are unstructured and highly disordered. Henceforth, to construct 3D model, we divided the model building steps into two parts: first, to build HD region and then its N-term. The C-term was not constructed, as the aim was to explore the localization of novel identified mutation D16N which is localized in the N-term.

Modeling of the homeodomain of *NKX2.5*

The crystal structures of the human HD, PDB-ID “3RKQ” (chain A, 137-194, resolution: 1.7 Å), “4S0H” (chain B, 138-194, resolution: 2.8 Å) and “5FLV” (chain A, 140-191, resolution 3.0 Å) were retrieved from protein data bank (www.rcsb.org). The target-templates multiple sequence alignment was carried out using ClustalW program, which is based on identifying structurally conserved regions (SCR) common to the template. The model was built through the Modeller v.-9.14 [5]. For each model the secondary structure comparisons was done by PSIPRED [6] program.

Building N-term model of *NKX2.5*

From the BLAST database, we failed to identify any protein structures (identification of template step) related to sequences of the N-term (from residue 1-137). The disorders prediction from DISOPRED server indicates that the N-term is highly unstructured and disordered. Therefore, we utilized comparative modeling strategy, shown as Scheme1: flowchart of molecular modeling pipeline (Figure 3), to build model for N-term region. Furthermore, the prediction of protein binding zone in the disordered N-term region was made by Anchor [7]. Apart from the PSIPRED, JPred [8] and RaptorX [9], tools were also used for the prediction of secondary structures in the disorder region of N-term. The fold recognition (FUGUE [10] and SAM-T06 [10]) and meta-threading and machine learning approach (eThread [11]) strategies were used for building the N-term region. Furthermore, to get the N-term model, the top models obtained from two different methods was compared with predicted secondary structure outcomes, and the consensus results which also matched with the result of ANCHOR (protein binding motifs regions) used as template for applying the comparative modeling strategy All models which have shown good DOPE-score, and Ramachndran plot were taken as template for model generation through Modeller v.-9.14(500 full-atom models). Now to merge the separately build, N-term and HD region, the templates of N-term and HD were used for further comparative modeling to get the final model (N-term + HD). In final models, the loop regions were refined using the LOOPY module of Jackal package [12]. Finally, the models were subjected to molecular dynamics simulations to remove bad contacts, steric clashes, and for optimization of hydrogens.

The biomolecular surface area, accessible to the solvent accessible surface area (SASA) [13] was performed. It is well known that hydrophobic amino acids, which are deeply buried in the protein core act as a driving force for folding of protein. Therefore, now a day, SASA used as a tool to distinguish correctly from incorrectly folded protein models. So according to this concept, in general the hydrophilic and polar amino acid should be exposed toward solvent and hydrophobic amino acid should be deeply buried and orient toward protein core.

The same revert well in our model as predicted through KNIME-Schrödinger [14].

Modeling of *GATA4*

The three-dimensional model of Zn-finger domain of human *GATA4* UniProt id.-P43694 (http://www.uniprot.org), was performed by comparative modeling strategy using the template structures of Zn-finger domain of *GATA4* (PDB-ID: 2M9W chain A), *GATA3* (PDB-ID: 4HC7 chain A, 3DFV chain C, 3DFX chain A, 3HC9 chain A, and 4HCA chain A), *GATA1* (PDB-ID: 3VD6 chain C, 1GAT chain A) and fungal *GATA* factor (PDB-ID: 4GAT chain A, 5GAT chain A). For the complete sequence, the percentage of sequence identity among *GATA* proteins was 100% (*GATA4*-vs-*GATA4*), 43% (*GATA4*-vs.-*GATA3*) and 39% (*GATA4*-vs.-*GATA1*), respectively. However, only for Zn-binding motif the sequence identity is 100%, 86%, 74%, while in case of yeast 58%. Though there is significant drop in identity while taking whole sequences of *GATA4*, but remarkable increase in identity was found when comparing only Zn-binding motifs. (Supplementary Figure 6).

The sequences alignment (with ClustalW) followed by model building through Modeller v.-9.14 (500 full-atom models). In each case, the best model was evaluated on the basis of DOPE-score, and Ramachandran plot was selected for further analysis. We used the ProSA program to check the fitness of the sequences relative to the obtained structures. The secondary structure predictions were performed similarly as in *NKX2.5*.

For further analysis of robustness and quality of modeled proteins (*NKX2.5* and *GATA4*, both), the ERRAT was used.

Finding of NKE motif in ANF promoter and Preparation of DNA

Human ANF-promoter nucleotide sequence genome seq. id FP000196 was retrieved from eukaryotic promoter database "EPD" (http://epd.vital-it.ch/). 600 bp 5'-flanking sequence was taken as ANF-promoter. All potential *NKX2.5* binding motif (NKE) in ANF promoter were retrieved by jasper (http://jaspar.binf.ku.dk/), and transfec database (http://www.gene-regulation.com/pub/databases.html) (Supplementary Figure 2B, Supplementary Table 1). In JASPER-database, we found 12 types of NKE-motif matrix model for NK2-family protein. Among 12, the four belongs to *NKX2.5* gene (3 form musmusculus, and 1 form c. elegans, Supplementary Figure 2B, Supplementary Table 1), however, no human *NKX2.5* NKE matrix model was found in JASPER database. The NKE-motifs are conserved throughout the evolution. [15, 16] All 4 NKE-motifs matrix model was used to screen the possible binding site/s of human *NKX2.5* of ANF-promoter DNA. The motif "AAGTG" (known to bind with *NKX2.5*) at -242bp was selected as a probe to build DNA and then DNA fragments of 20-20 nucleotides ±41 residue were taken. DNA was constructed by 3D-DART [17] and "supercomputing facility for bioinformatics& computational biology, IIT Delhi (http://www.scfbio-iitd.res.in/software/drugdesign/bdna.jsp#)" webservers.

Protein-DNA docking

NKX2.5-DNA complex was formed by two-way. First, the DNA was taken from published crystal structure of *NKX2.5-ANF* complex PDB-ID "3RKQ" and "4S0H" and the complex with best minimized model was formed by overlay of the HD region and crystal of *NKX2.5*. Formed complex was further energy minimized to remove bad clashes. Secondary, the protein-DNA docking was performed through web servers HADDOCK2.2 [18] and NPDock [19]. The best complex was screened out by taking as standard, interaction of HD of *NKX2.5* with DNA as reported in PDB ID: 3RKQ (Supplementary Figure 2A).

Protein-Protein Docking (P-P docking) NKX2.5-GATA4

An extensive multi-step protein-protein docking between best models of *GATA4* and of *NKX2.5* was performed by SWARMDOCK [20] and Maestro/PIPER (Schrödinger Inc.) tool [21]. The docking outcomes were separately evaluated to get the best complexes. Furthermore, the focused docking was conducted to get the proper orientation of the complexes (i.e binding mode analysis). However, to get more robust outcome and to validate the result of protein-protein docking we further performed the SiteMap [22] analysis through Schrodinger module.

Role of Mutation: We mutate D16 of *NKX2.5* into N16 and relax the protein by using OPLS3 force field [23] in Schrödinger suite (Schrödinger Release, 2017-1) [24] the final protein is now *NKX2.5* MT. The mutated protein was superimposed over wild type, to check the variability in the interaction map.

RESULTS

Modeling of NKX2.5:

Construction of homeodomain (HD)

NKX2.5 HD adopts a unique canonical form of architecture, which is specific to HD containing proteins acting as transcription factor (Figure 5). The best 3D model for HD (from amino acid residue 141-192) of *NKX2.5* has been obtained using as template structures i.e. the PDB-IDs: "3RKQ" (chain a, 137-194, resolution:

1.7 Å) [25], "4S0H" (chain b, 138-194, resolution: 2.8 Å) [26] and "5FLV" (chain a, 140-191, resolution 3.0 Å) [27]. The Final model of HD has 97.8% residues in the most favored regions in Ramachandran plot, Prosa Z-score of -4.54 and Errat overall quality factor of 91.7 while, template 4S0H has 92.0% residue in the most favored region in Ramachandran plot (Supplementary Figure 3B), Prosa Z-score of -4.57 (Supplementary Figure 4B) and Errat overall quality factor of 98.0 (Supplementary Figure 5C). The HD zone appears well structured as in agreement with its low propensity to the disorder (Figure 1E). The 3D homology model of HD matches very well with the secondary structure predictions and the architecture of secondary structure in 3-dimensional space completely mimic the published crystals "3RKQ" and "4S0H". The Dope score of model was showing the same trend as in crystals (Supplementary Figure 9A). The C-alpha RMSD analysis between HD region of final model of *NKX2.5* and its template is 0.345Å, indicating the robustness and good quality of the model.

Construction of N-term region

The N-term region is positioned above the DNA binding site of HD in such a manner so that it makes a lid like structure, probably N-term regulates the on/off switch behavior. N-term model has 86.7% residues in the most favored regions in Ramachandran plot (Supplementary Figure 3C) and a Prosa Z-score of 4.28 (Supplementary Figure 4C). The solvent accessibilitycalculation (SASA) was carried out to further validate the model (Supplementary Figure 6A). Final model falls in the line with the result of SASA as the exposed residues lied at outer face of model away from the core/grove and exposed to solvent, while the deep and buried residues form hydrophobic inner globular core.

Modeling of *GATA4*

Human *GATA4* is broadly divided into three big domains, viz. N-term, CORE and C-term. The N-term have two small Transcriptional activation domain (TAD), core contain two Zn-finger (ZnF) of the motif Cys-X2-Cys-X17-Cys-X2-Cys that directs binding to the nucleotide sequence element (A/T) GATA (A/G). [28] The C-term also has one large TAD. The *GATA4* model (214-326) made up of N-term region (210-213), ZnF1-domain (214-247), connecting loop (248-268), ZnF2-domain (269-302), C-term region (303-326) (Supplementary Figure 1C) [29]. Each ZnF made up of one helix of 10 amino acid and two anti-parallel beta sheets. (Figure 6B and S8) From the Figure 1F it is clear that the *GATA4* have two strong protein binding zones one is localized at the in C-term (from amino acid residue 300-420), and other is at the center between two Zn-Finger residues 248-268. However, at N-term, either very weak or no any protein binding region is identified.

Multiple sequence alignment of human GATA family proteins human GATA: GATA1 to GATA6, and mouse GATA: GATA1, GATA3 and *GATA4*; and from the crystal structures template PDBs, it was clear that core region from residue 214 to 326 remain highly conserved throughout its the family. However, while opposite to this very less similarity was found at in N-term and C-term regions. (Supplementary Figure 6B).

The best 3D model of core region has been obtained by using as crystal structures (template PDB ID: "4HC7" (chain a, 259-366, GATA3-complex2, resolution: 2.7 Å)[3]; "3VD6" (chain c, 200-318, 1.9 Å)[3]; "4HC9" (chain a, 257-371, GATA3-complex1, resolution: 1.6 Å)[4]; "4HCA" (chain a, 257-371, resolution 2.8 Å) and "2M9W" (chain a, 261-321, *GATA4*) shown in Supplementary Figure 9. This model of core region has 99.9% residues in the most favored regions in Ramachandran plot and a Prosa Z-score of 8.7. These values, compared also with those of the template structures (including ERRAT values, Supplementary Figure 5G and Dope_score, Supplementary Figure 9B), indicating the quality of the model.

Disorder, globularity and binding region prediction in *GATA4*

Similar to *NKX2.5* the disorderness was also calculated for *GATA4*. As shown in Figure 1F that the amino acid residue from 1 to 170 and 320 to 425 are highly disordered (confidence score >0.8), residue 170 to 210 are moderate disordered (confidence score 0.6-0.8), residue 210 to 320 and 425 to 442 are structured (confidence score <0.5). The confidence score per residue-wise, in which the confidence score above 0.5 is counted as disordered region. From the Figure 1F it is clear that, the *GATA4* have two strong protein binding sites, one localized in C-term disorder part (from residue 300-420) and, second is at connecting loop i.e between two ZnF-domains (residues 248-268). The globularity prediction (Figure 1F) also matched with DISOPRED and IUPRED findings that the region beyond core region (ZnF domains) is non-globular and highly flexible. So here we consider only ZnF domain regions for model building. Similar to NKX, the best model was evaluated on the basis of Prosa score which -4.59 (4HCA), -4.47 (4HC7) and -4.78 (model). We further crosschecked the robustness of *GATA4* model by doing ERRAT analysis. The ERRAT values 90.0 (4HCA), 73.6 (4HC7) and 79.8 (model) (Supplementary Figure 7). These values were supported well with ramachandran scores which is 70.4% (4HCA), 77.5% (4HC7) and 91.8% (model). (Supplementary Figure 4) Furthermore the Dope_ score and RMSD values were performed. The Dope_score of HD regions and its model follow similar behavior, and

their RMSD value with template is 1.77. These multi-step modeling analyses claimed the robustness of our model.

Explanation of ERRAT

Although ERRAT is very effective in identifying erroneous and non-erroneous regions in model structure but have one weakness of higher sensitivity toward small deviations in atomic positions. It means those regions in protein which are flexible or have high "R" value, disordered or involved in disorder-to-order transition (during interaction with other partner proteins) have high error value, and because of this, although the structure of protein is compact, though the overall quality factor decreased significantly. It is well evident in case of human *GATA3*, PDB ID: 4HCA and 4HC7 (from 260-369, resolution 2.80 Å). In both PDBs, the residue 304-316 has shown higher disorder tendency (> 0.5) (Figure 1F). This region is flexible enough, as in 4HCA it is missing, while in 4HC7, it has shown very high B-factor. The flexibility and disorderedness impact the overall quality of proteins as in 4HC7, the calculated overall quality of is 73.6% (error value 99.0%), while in 4HCA it is 90% (Supplementary Figure 7E and 7F). Furthermore, the same analysis was performed on *GATA4* model, where the region to be address are localized from residue 257-270. This region also has shown the disordered tendency > 0.5. The overall quality factor for model *GATA4* is 79.9 (error value 99.0%) (Supplementary Figure 7G) which is considerably higher value than crystal 4HC7, indicating the reliability of our constructed model.

From this discussion, it is convincible that the regions in protein which are disordered have high error value in ERRAT score. In *NKX2.5*, the crystal information is available for HD region only. The overall quality factor for HD region is 91.7, which is near to its crystal structure, PDB: 4S0H (94.8). In the final model of *NKX2.5* (N-term + HD), the overall quality factor decreased enormously (61.1). The abrupt drop of quality is due to disordered N-term region of *NKX2.5* (Supplementary Figure 7D).

REFERENCES

1. Chung IM, Rajakumar G. Genetics of congenital heart defects: The NKX2-5 gene, a key player. Genes. 2016; 7: 6.
2. Apergis GA, Crawford N, Ghosh D, Steppan CM, Vorachek WR, Wen P, Locker J. A novel nk-2-related transcription factor associated with human fetal liver and hepatocellular carcinoma. Journal of Biological Chemistry. 1998; 273: 2917-25.
3. Ward JJ, McGuffin LJ, Bryson K, Buxton BF, Jones DT. The DISOPRED server for the prediction of protein disorder. Bioinformatics. 2004; 20: 2138-9.
4. Dosztányi Z, Csizmok V, Tompa P, Simon I. IUPred: web server for the prediction of intrinsically unstructured regions of proteins based on estimated energy content. Bioinformatics. 2005; 21: 3433-4.
5. Webb B, Sali A. Protein structure modeling with MODELLER. Protein Structure Prediction. 2014: 1-15.
6. McGuffin LJ, Bryson K, Jones DT. The PSIPRED protein structure prediction server. Bioinformatics. 2000; 16: 404-5.
7. Dosztányi Z, Mészáros B, Simon I. ANCHOR: web server for predicting protein binding regions in disordered proteins. Bioinformatics. 2009; 25: 2745-6.
8. Cuff JA, Clamp ME, Siddiqui AS, Finlay M, Barton GJ. JPred: a consensus secondary structure prediction server. Bioinformatics. 1998; 14: 892-3.
9. Wang S, Li W, Liu S, Xu J. RaptorX-Property: a web server for protein structure property prediction. Nucleic acids research. 2016; 44: W430-W5.
10. Shi J, Blundell TL, Mizuguchi K. FUGUE: sequence-structure homology recognition using environment-specific substitution tables and structure-dependent gap penalties. Journal of molecular biology. 2001; 310: 243-57.
11. Brylinski M, Feinstein W. Setting up a meta-threading pipeline for high-throughput structural bioinformatics: eThread software distribution, walkthrough and resource profiling. J Comput Sci Syst Biol. 2012; 6: 001-10.
12. Xiang JZ, Honig B. Jackal: A protein structure modeling package. Columbia University and Howard Hughes Medical Institute, New York. 2002.
13. Durham E, Dorr B, Woetzel N, Staritzbichler R, Meiler J. Solvent accessible surface area approximations for rapid and accurate protein structure prediction. Journal of molecular modeling. 2009; 15: 1093-108.
14. Warr WA. Scientific workflow systems: Pipeline Pilot and KNIME. Journal of computer-aided molecular design. 2012; 26: 801-4.
15. Durocher D, Charron F, Warren R, Schwartz RJ, Nemer M. The cardiac transcription factors Nkx2-5 and GATA-4 are mutual cofactors. EMBO J. 1997; 16: 5687-96.
16. Durocher D, Chen CY, Ardati A, Schwartz RJ, Nemer M. The atrial natriuretic factor promoter is a downstream target for Nkx-2.5 in the myocardium. Mol Cell Biol. 1996; 16: 4648-55.
17. van Dijk M, Bonvin AM. 3D-DART: a DNA structure modelling server. Nucleic acids research. 2009; 37: W235-W9.
18. Van Zundert G, Rodrigues J, Trellet M, Schmitz C, Kastritis P, Karaca E, Melquiond A, Van Dijk M, De Vries S, Bonvin A. The HADDOCK2. 2 web server: user-friendly integrative modeling of biomolecular complexes. Journal of molecular biology. 2016; 428: 720-5.

19. Tuszynska I, Magnus M, Jonak K, Dawson W, Bujnicki JM. NPDock: a web server for protein–nucleic acid docking. Nucleic Acids Res. 2015; 43:W425-30.

20. Torchala M, Moal IH, Chaleil RA, Fernandez-Recio J, Bates PA. SwarmDock: a server for flexible protein–protein docking. Bioinformatics. 2013; 29: 807-9.

21. Kozakov D, Brenke R, Comeau SR, Vajda S. PIPER: an FFT-based protein docking program with pairwise potentials. Proteins: Structure, Function, and Bioinformatics. 2006; 65: 392-406.

22. Schrödinger Release 2017-1: SiteMap. New York (NY): Schrödinger, LLC; 2017.

23. Harder E, Damm W, Maple J, Wu C, Reboul M, Xiang JY, Wang L, Lupyan D, Dahlgren MK, Knight JL, Kaus JW, Cerutti DS, Krilov G, et al. OPLS3: a force field providing broad coverage of drug-like small molecules and proteins. J Chem Theory Comput. 2016; 12:281–96.

24. Small-Molecule Drug Discovery Suite 2017-2. New York (NY): Schrödinge r, LLC. 2017.

25. Pradhan L, Genis C, Scone P, Weinberg EO, Kasahara H, Nam HJ. Crystal structure of the human NKX2. 5 homeodomain in complex with DNA target. Biochemistry. 2012; 51: 6312-9.

26. Pradhan L, Gopal S, Li S, Ashur S, Suryanarayanan S, Kasahara H, Nam HJ. Intermolecular Interactions of Cardiac Transcription Factors NKX2.5 and TBX5. Biochemistry. 2016; 55: 1702-10.

27. Luna-Zurita L, Stirnimann CU, Glatt S, Kaynak BL, Thomas S, Baudin F, Samee MA, He D, Small EM, Mileikovsky M, Nagy A, Holloway AK, Pollard KS, et al. Complex Interdependence Regulates Heterotypic Transcription Factor Distribution and Coordinates Cardiogenesis. Cell. 2016; 164: 999-1014.

28. Chen Y, Bates DL, Dey R, Chen PH, Machado ACD, Laird-Offringa IA, Rohs R, Chen L. DNA binding by GATA transcription factor suggests mechanisms of DNA looping and long-range gene regulation. Cell reports. 2012; 2: 1197-206.

29. Gallagher JM, Yamak A, Kirilenko P, Black S, Bochtler M, Lefebvre C, Nemer M, Latinkić BV. Carboxy terminus of GATA4 transcription factor is required for its cardiogenic activity and interaction with CDK4. Mechanisms of development. 2014; 134: 31-41.

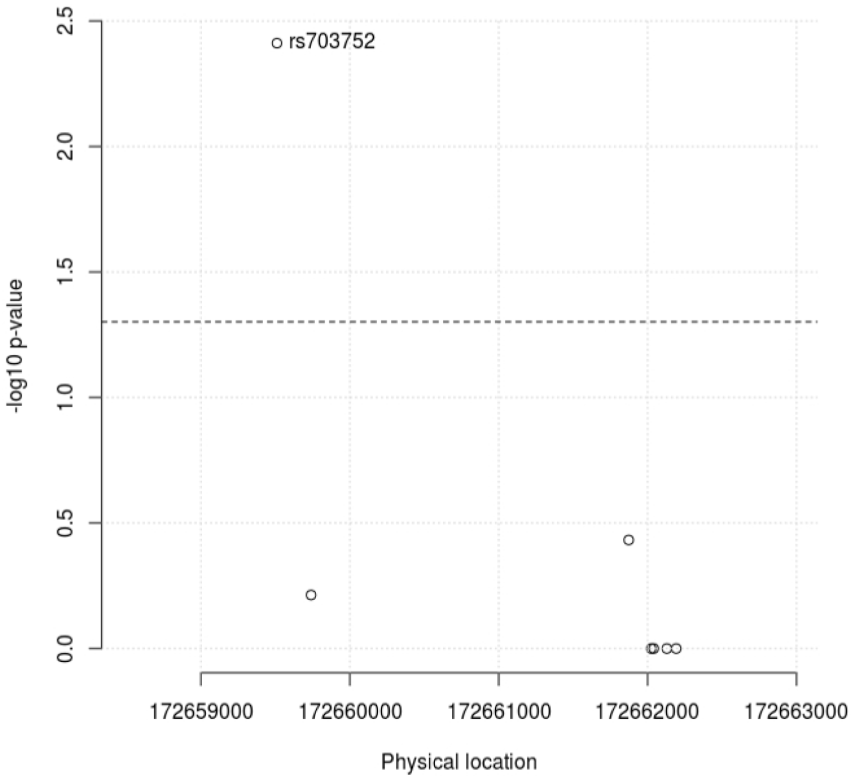

Supplementary Figure 1: Hardy-Weinberg equilibrium P-value.

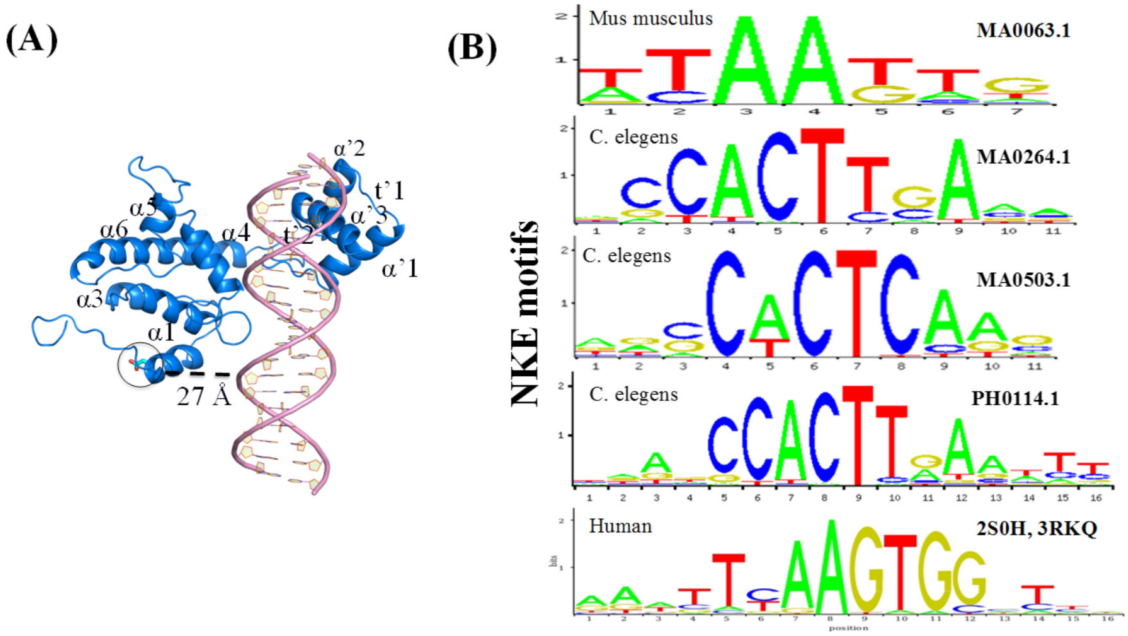

Supplementary Figure 2: (A) Overall structure of *NKX2.5* (only N-term + HD) and ANF-242 complex. The *NKX2.5* is shown in blue color, and the DNA is shown in pink. The residue D16 of *NKX2.5 is* shown in stick representation (in circle) with carbon in cyan, oxygen in red and nitrogen in blue colour. Identified mutation residue D16 has distance (27Å) and no any interaction with DNA. **(B)** *NKX2.5* binding motif in ANF-242 DNA.

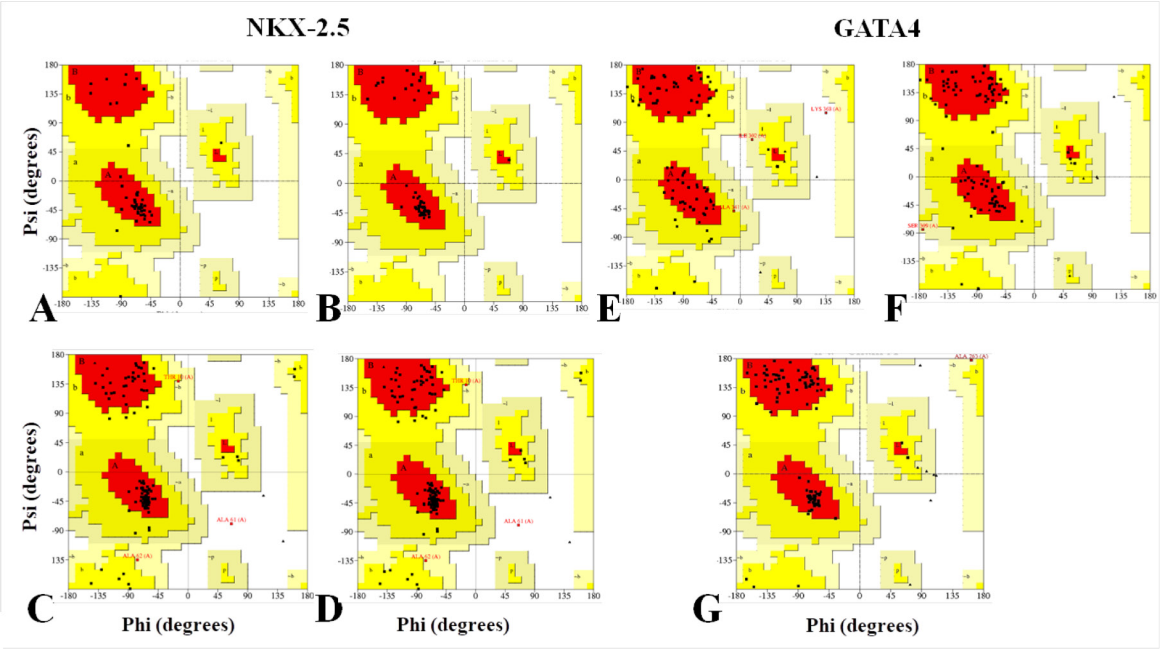

Supplementary Figure 3: Structure validation results of *NKX2.5* and *GATA4* by Ramachandran plot: Ramachandran analysis of the backbone dihedral angles PSI (ψ) and PHI (ϕ) for *NKX2.5* **(A)** Template "4S0H", **(B)** model HD, **(C)** model N-term **(D)** complete model (N-term + HD). For *GATA4* templates **(E)** "4HC7", **(F)** "4HCA", **(G)** model *GATA4*.

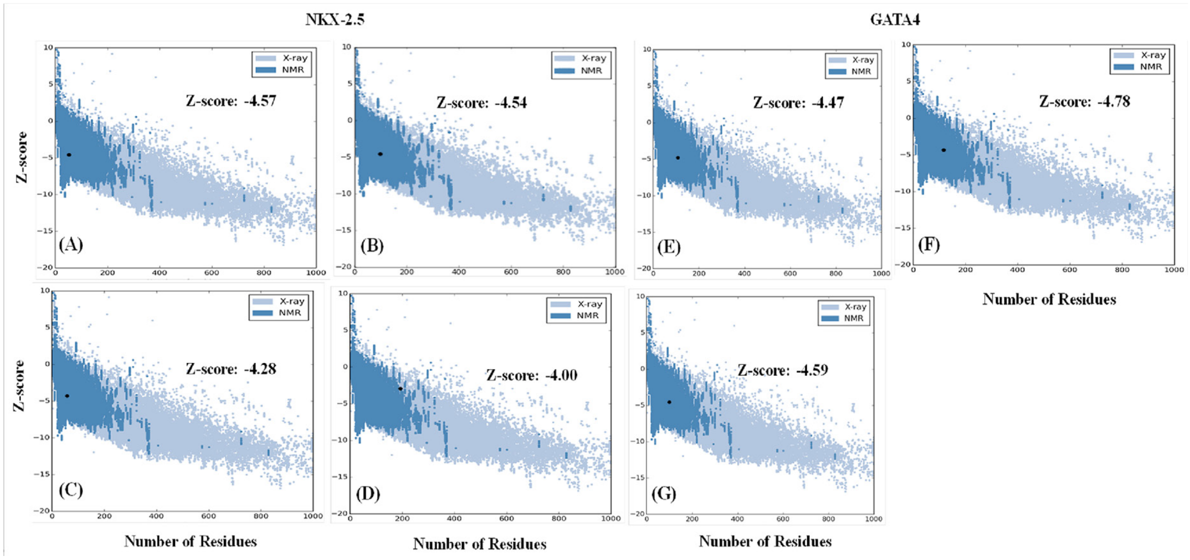

Supplementary Figure 4: Structure validation results of *NKX2.5* and *GATA4* by Prosa Z_score verification tool: Prosa Z_score plots of *NKX2.5* and *GATA4*: The Z-score of modeled proteins are represented as black dots. For *NKX2.5* **(A)** template "4S0H", **(B)** model HD-*NKX2.5*, **(C)** model N-term, **(D)** complete model (N-term + HD). For *GATA4* **(E)** template "4HC7", **(F)** template "4HCA", **(G)** model *GATA4*.

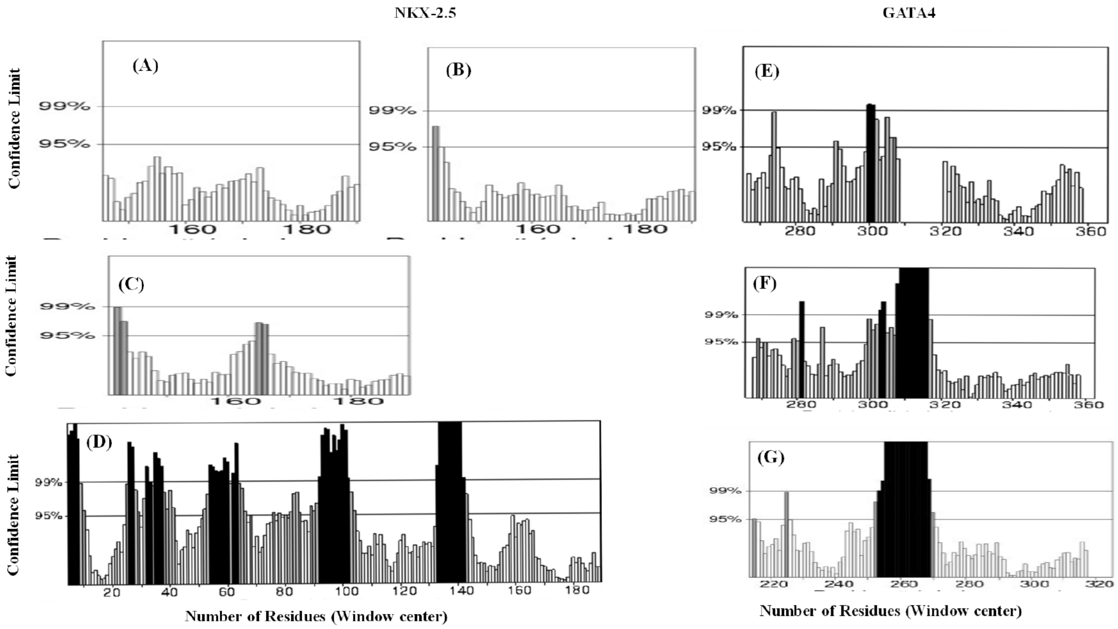

Supplementary Figure 5: Structure validation results of *NKX2.5* and *GATA4* by ERRAT verification tool: for *NKX2.5* templates **(A)** "4S0H", **(B)** "3RKQ", **(C)** model HD-*NKX2.5*, **(D)** for complete model (N-term + HD). For *GATA4* templates **(E)** "4HC7", **(F)** "4HCA", **(G)** model *GATA4*.

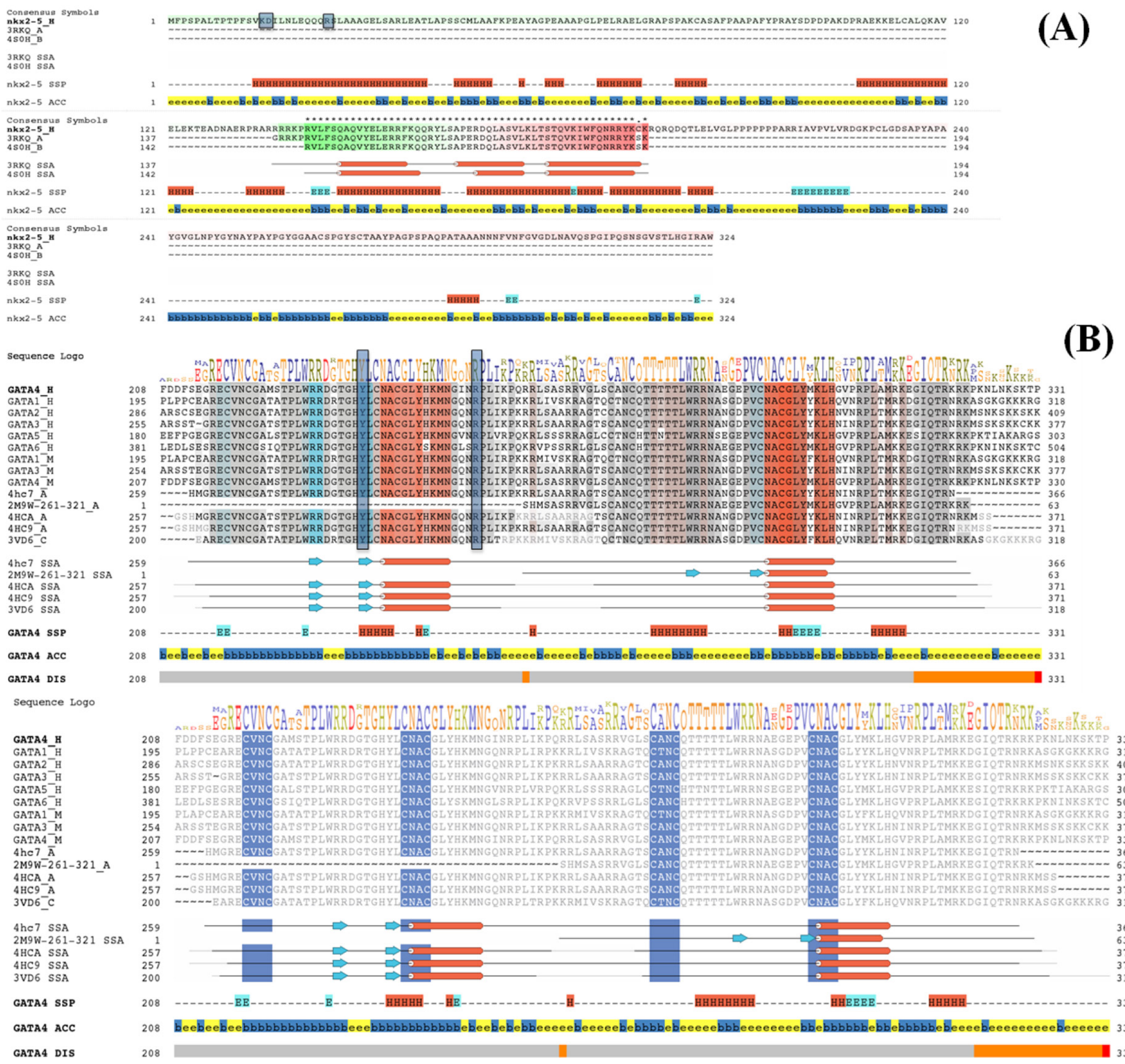

Supplementary Figure 6: Conserveness, secondary structure analysis and solvent accessibility zones of *NKX2.5* and *GATA4*: Consensus residue is marked via symbol "*". Secondary structure prediction was done through PSIPRED. The helices are marked "H", and "E" marks beta sheets. In solvent accessibility prediction, the residues exposed to the solvent are marked as "e" yellow colour (more than 25% of total surface area is predicted to be exposed to the solvent), while residue deep buried is marked "b" in blue color. **(A)** Complete sequence of *NKX2.5* aligned with template PDB-IDs "3RKQ" and "4S0H" and residue wise prediction of secondary structure and solvent accessibility. **(B)** *GATA4* Zn-binding region aligned with templates and residue wise prediction of secondary structure and solvent accessibility.

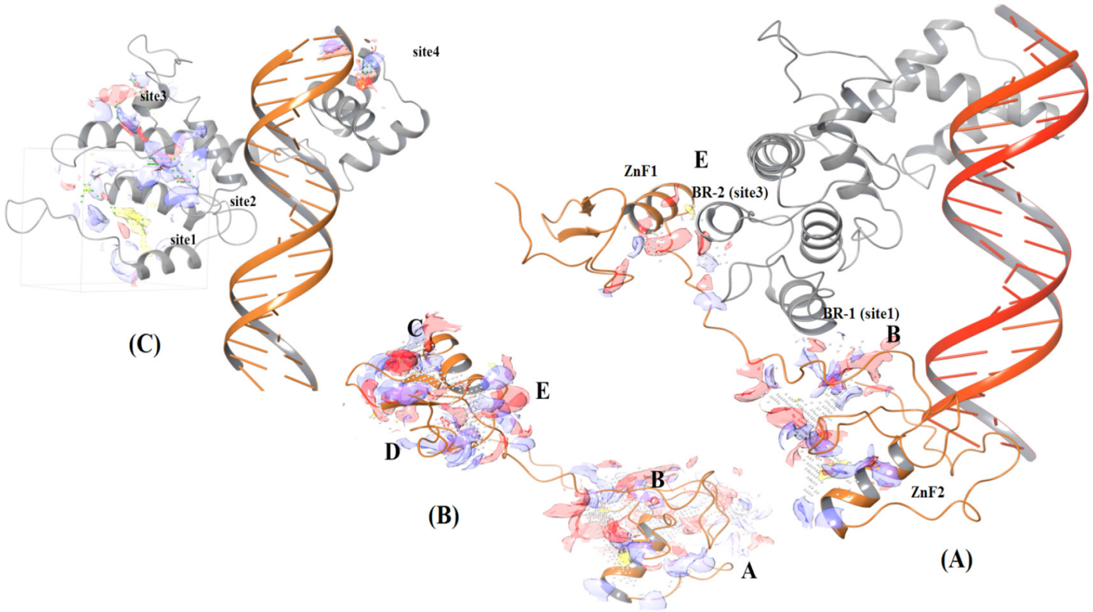

Supplementary Figure 7: The interface site between *NKX2.5* and *GATA4*: the binding site search was performed through Schrodinger. The red and blue clouds are acidic and basic residues, respectively.

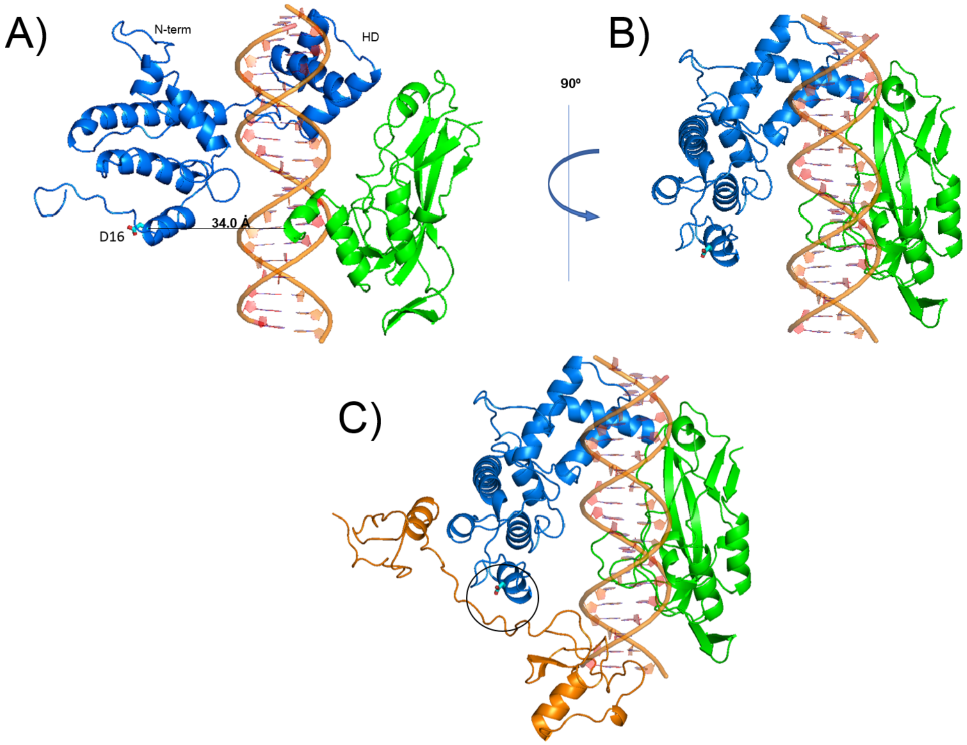

Supplementary Figure 8: ***NKX2.5, GATA4, TBX5 and DNA*** **assembly:** NKX2.5-DNA-GATA4-TBX assembly. DNA (ANF-promoter) light orange colour, NKX2.5 in dark blue, TBX5 in green colour, GATA4 in deep orange colour and amino acid D16 (stick representation, at N-term of NKX2.5) shown in cyan colour. **(A)** NKX2.5-DNA-TBX5 complex, C-alpha atom of D16 amino acid is very distant from TBX5 (34.0 Å) **(B)** NKX2.5-DNA-TBX5 complex after 90º rotation, **(C)** NKX2.5-DNA-GATA4-TBX5 assembly. The interaction region of D16 with GATA4 shown by dark circle.

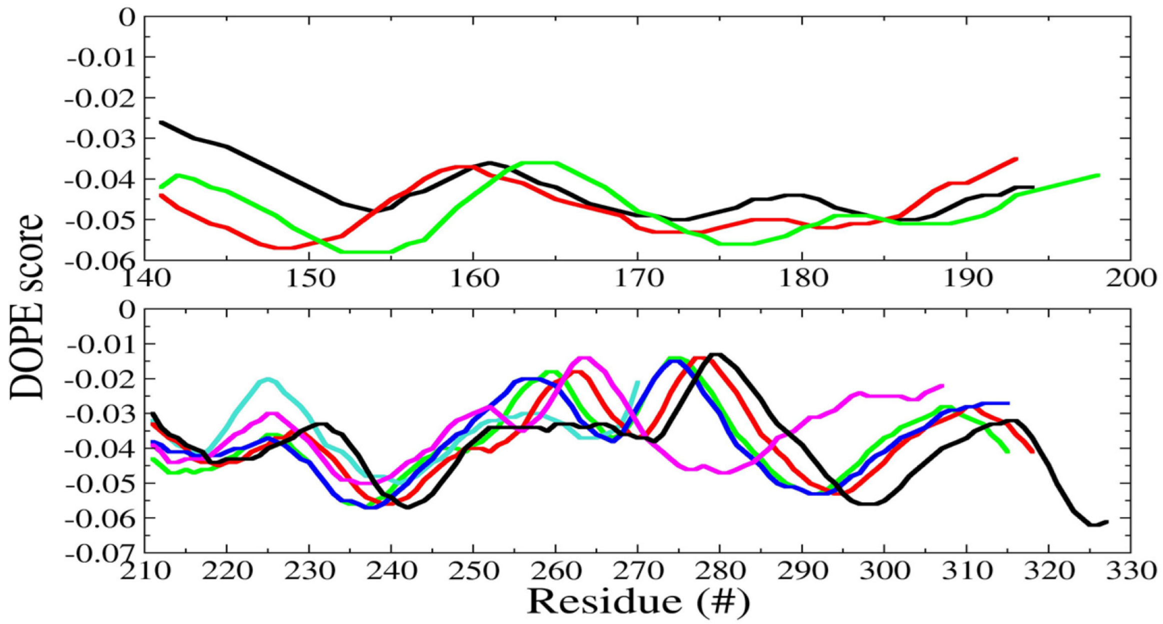

Supplementary Figure 9: Structure validation results of *NKX2.5* and *GATA4* by Dope_score analysis: Discrete optimized potential energy (DOPE) score profiles of the templates and modeled *NKX2.5-HD* (upper panel) and *GATA4* (lower panel).

Supplementary Table 1: *NKX2.5* binding motif in ANF promoter

S. No.	Model ID	Model name	Score	Relative score	Start	End	Strand	predicted site sequence
1	MA0503.1	*NKX2.5*(var.2)	7.532	0.86888232	62	72	1	ctcctctccac
2	MA0063.1	NKX2.5	4.994	0.8259446219	72	78	-1	ataaggg
3	MA0063.1	NKX2.5	5.189	0.8337954887	75	81	1	ttatttg
4	PH0111.1	Nkx2-2	8.592	0.8018165371	235	251	-1	gctcccacttcaaaggt
5	MA0063.1	NKX2.5	5.092	0.8298901857	240	246	1	tg$_{242}$aagtg
6	MA0264.1	ceh-22	9.043	0.8555266753	250	260	1	gcctcttgagt
7	MA0063.1	NKX2.5	6.122	0.8713588667	252	258	-1	tcaagag
8	MA0503.1	*NKX2.5*(var.2)	4.864	0.8295840037	288	298	-1	agaccctcagc
9	MA0063.1	NKX2.5	4.64	0.8116922791	362	368	1	ttaaaag
10	MA0063.1	NKX2.5	5.075	0.8292057512	409	415	1	gc$_{80}$aagtg
11	MA0503.1	*NKX2.5*(var.2)	6.467	0.8531953984	441	451	-1	aagcacgcagg
12	MA0503.1	*NKX2.5*(var.2)	7.534	0.868911779	450	460	-1	cagctctccaa
13	MA0503.1	*NKX2.5*(var.2)	2.92	0.8009498483	517	527	-1	cccctctcttg
14	MA0503.1	*NKX2.5*(var.2)	6.028	0.8467291462	530	540	-1	ttcctctctgg
15	MA0503.1	*NKX2.5*(var.2)	3.21	0.8052214044	569	579	-1	gagcaatccac
16	MA0503.1	*NKX2.5*(var.2)	3.576	0.8106124028	638	648	1	tggcattccag

Transfec server predicts total 13 putative sites with Relative profile score threshold set to 80%. The place marked in yellow show best motif.

Supplementary Table 2: Polar-Interaction of *NKX2.5*-DNA complex (<4.0 Å)

Types of Intera-ction	*NKX2.5*WT-DNA			*NKX2.5*MT-DNA		
	*NKX2.5*WT	DNA	Distance (Å)	*NKX2.5*MT	DNA	Distance (Å)
Protein-DNA	R142:NH1	G4:N3	3.3	R142:NH1	G4:N3	3.3
	R142:NH1	A5:O4	2.4	R142:NH1	A5:O4	2.4
	R142:NH2	A9':O4	2.6	R142:NH2	A9':O4	2.6
	Y162:OH	A3':O1P	4	Y162:OH	A3':O1P	4
	Q181:NE2	A6:O1P	4.6	Q181:NE2	A6:O1P	4.6
	Q187:NE2	C4':1H4	2.7	Q187:NE2	C4':1H4	2.7
	Q187:NE2	A3':2H6	2.6	Q187:NE2	A3':2H6	2.6
	Q187:NE2	A3':N7	2.9	Q187:NE2	A3':N7	2.9
	Q187:OE1	A3':H8	4	Q187:OE1	A3':H8	4
	N188:OD1	A6:2H6	2	N188:OD1	A6:2H6	2
	N188:ND2	A6:N7	3.1	N188:ND2	A6:N7	3.1
	R190:NH1	A3':O2P	4	R190:NH1	A3':O2P	4
	Y191:OH	T5':O2P	3.1	Y191:OH	T5':O2P	3.1
	K192:NZ	G4:O2P	3.2	K192:NZ	G4:O2P	3.2
	K194:NZ	A3:OP2	4.4	K194:NZ	A3:OP2	4.4

WT= wild type, MT= mutant type.

Residue in DNA 5' chain shown in regular numbers, While in 3' shown with complementary numbers.

Supplementary Table 3: Polar interactions between *NKX2.5* and *GATA4* complex

MOTIFs	Hydrogen Bonds (<3.5 Å)					
	NKX2.5WT-*GATA4*			*NKX2.5*MT-*GATA4*		
	NKX2.5	*GATA4*	Distance (Å)	*NKX2.5*	*GATA4*	Distance (Å)
Binding Region 2(BR2)	Q22:NE2	Q258:O	3.2	Q22:NE2	Q258:O	3.4
	N19:ND2	Q258:O	3.5	N19:ND2	Q258:O	3.8
	K15:NZ	R259:O	2.6	K15:NZ	R259:O	2.9
	K15:NZ	R260:O	2.7	R260:O	NT	NT
	D16:OD2	R260:NH1	3.2	R260:NH1	NT	NT
Binding Region 1(BR1)	Q32:OE2	R252:NH1	3.4	Q32:OE2	R252:NH1	3.7
	Q32:OE1	R252:NH2	3.4	Q32:OE1	R252:NH2	3.6
	R25:NH2	Y244:OH	3.1	R25:NH2	Y244:OH	NT
	R36:NH1	R252:NH2	3.6	R36:NH1	R252:NH2	3.6
	R36:NH2	R252:NH2	4.1	R36:NH2	R252:NH2	4.1

Only below 4.0 Å interactions are counted.

Supplementary Table 4: Sitemap prediction of binding sites:

Supplementary Table 4 (A): *GATA4*

Site map site-no.	Site Score	Size	Dscore	Volume (a)	Expo-sure	Enclo-sure	Contact	Phobic (php)	Philic (phl)	Balance (php/phl)	Don/ acc
A	0.885	419	0.982	183.848	0.679	0.403	0.378	0.095	0.614	0.154	0.633
B (motif-1)	0.878	254	0.997	99.127	0.782	0.353	0.323	0.128	0.473	0.271	0.626
C	0.87	229	0.985	100.499	0.745	0.349	0.331	0.083	0.506	0.163	0.772
D	0.721	69	0.809	34.643	0.871	0.303	0.262	0.064	0.47	0.135	1.04
E (motif-2)	0.668	55	0.742	19.551	0.898	0.309	0.277	0.08	0.43	0.185	0.223

Supplementary Table 4 (B): *NKX2.5*

Site map site-no.	Site Score	Size	Dscore	Volume (a)	Expo-sure	Enclo-sure	Contact	Phobic (php)	Philic (phl)	Balance (php/phl)	Don/ acc
1	0.847	50	0.885	194.138	0.766	0.63	0.745	1.41	0.484	2.911	1.816
2	0.771	54	0.597	137.886	0.665	0.646	0.812	0	1.478	0	1.841
3	0.699	43	0.641	128.625	0.707	0.626	0.882	0.21	1.076	0.195	0.641
4	0.662	28	0.595	73.402	0.622	0.686	0.926	0.657	0.962	0.683	0.581

(A) in *GATA4*, the surface clefts, A, B, C, D and E. Site A (Site score, 0.89; Dscore, 0.98), largest in size and occur at PB'1 of*GATA4* which interact with C-term of *NKX2.5*. 2. Site B (Sitescore, 0.88; Dscore, 1.0; balance 0.271), extended from residue R259-K300, and occupy the top face of ZnF2 and close to BR1 in *NKX2.5*. Site E, (Site score 0.67; Dscore, 0.74; balance, 0.19) occupy the place of interaction zone of BR2, side face of ZnF1. It is interesting to note that site B and E respectively matched with BR1 and BR2 of interacting sites. **(B)** in *NKX2.5*, **Site1** (Site score, 0.85; Dscore, 0.89), largest in size and occur at BR1, placed near to novel mutation D16N. Although this site has higher balance score>1.6, means very tight binding, but when keenly observed as shown in image we found that the interacting surface which participate in interaction with *GATA4* are hydrophilic. **Site4**, occur in HD region near the PB2 which have interaction with *TBX5*. **Site3** cover BR2 and **Site2** partially cover both BR1 and BR2, but as it is clear cut no any protein binding zone detected separately for Site3 and Site2.

Supplementary Table 5: Amino acid residues of *NKX2.5* (HD) present in cutoff value 8.0 Å from DNA (cut off taken through pymol)

MOTIFs	NKX2.5				
	Residues (8.0A)	POLAR interactions (PI)	Mutation (MT)	PTM	Total mutation in HD (17)
Homeodomain (HD)-DNA interaction	R142	R142	R 142 C		R 142 C
	V143				Q149Term
	L144				V 150 I
	F145				Q 160 P
	V150		V 150 I		R 161 P
	Y162	Y162			Q170Term
	S164			S164	L 171 P
	A165				T 178 M
	T180			T180	S 179 P
	Q181	Q181	Q 181 Q		Q 181 Q
	K183				
	I184				
	W185		W 185 L		W 185 L
	Q187	Q187	Q 187 H		Q 187 H
	N188	N188	N 188 K		N 188 K
	R189		R189 G		R 189 G
	R190	R190	R 190 H/L/C		R 190 H/L/C
	Y191	Y191	Y 191 C		Y 191 C
	K192	K192			Q198Term
	K194	K194			

All categories, **(A)** only interaction, **(B)**. Interaction with mutation**, (C)**. Interaction with PTMs and **(D).** only mutations are listed.

Supplementary Table 6: Results summary for Change in binding free energy/affinity (ΔΔGb) (kcal/mol) of NKX2.5-GATA4 complex system after mutating the residues to known mutant and to alanine residue

Residue	Mutation to Known Mutant	Change in binding affinity (ΔΔGb Kcal/mol)		Mutation to Alanine	Change in binding affinity (ΔΔGb Kcal/mol)	
		BioLuminate	Beatmusic		BioLuminate	Beatmusic
**NKX2.5-MUTATION**						
LYS15	K → I	-7.31	0.86	K → A	-3.07	0.92
**ASP16**	**D → N**	**5.01**	0.85	D → A	8.65	0.68
ASN19	N → S	3.85	0.75	N → A	3.59	1.33
GLU21	E → Q	2.12	0.08	E → A	2.09	0.04
GLN22	Q → K	2.86	0.28	Q → A	2.58	0.43
ARG25	R → C	2.95	0.42	R → A	2.95	0.44
GLU32	E → K	7.65	1.29	E → A	7.44	0.99
ARG36	R → S	-2.92	0.52	R → A	-2.26	0.57
**GATA4-MUTATION**						
ASN239	N → D	0.91	0.14	N → A	0.1	-0.03
TYR244	Y → C	2.58	1.62	Y → A	3.61	1.72
MET247	M → T	4.24	0.59	M → A	5.28	0.51
ASN248	N → S	1.19	0.58	N → A	1.79	0.43
ARG252	R → P	1.65	1.17	R → A	2.18	1.41
ILE255	I → T	2.16	0.25	I → A	1.98	0.52
ARG260	R → Q	10.55	0.74	R → A	16.32	1.77
LEU261	L → P	0.77	0.26	L → A	0.53	-0.06

ΔΔGb value higher than zero means decrease in binding affinity and value less than zero means increase in binding affinity.

Supplementary Table 7: List of primers used for amplification and sequencing of *NKX2.5*

S.No.	Primer	Primer Sequence	Annealing Temp	Product length
**1**	S1 *NKX2.5*_h	CTGCTCATCGCTCCTGTCATCGAG	62	721
**2**	AS1 *NKX2.5*_h	GGCGACAACACCAGGCATCTTACA		
**3**	S2 *NKX2.5*_h	TTCAGTTCACCCCAGAGCATCACCT	62	1014
**4**	AS2 *NKX2.5*_h	CTTCCCTACCAGGCTCGGATACCAT		
**5**	S3 *NKX2.5*_h	GGGGACTTGAATGCGGTTCAGAGC	63	723
**6**	AS3 *NKX2.5*_h	ATCTCAGAAAGTGCCCGACACGGA		
